# Site-Selective Modification of a Porpholactone—Selective Synthesis of 12,13- and 17,18-Dihydroporpholactones

**DOI:** 10.3390/molecules25112642

**Published:** 2020-06-06

**Authors:** Ana F. R. Cerqueira, Gustautas Snarskis, Jonas Zurauskas, Samuel Guieu, Filipe A. Almeida Paz, Augusto C. Tomé

**Affiliations:** 1 LAQV-REQUIMTE, Department of Chemistry, University of Aveiro, 3810-193 Aveiro, Portugal; anacerqueira@ua.pt (A.F.R.C.); gustautass@gmail.com (G.S.); jonas.zurauskas@gmail.com (J.Z.); sguieu@ua.pt (S.G.); 2 CICECO–Aveiro Institute of Materials, Department of Chemistry, University of Aveiro, 3810-193 Aveiro, Portugal; filipe.paz@ua.pt

**Keywords:** porpholactones, dihydroporpholactones, 1,3-dipolar cycloadditions, pyrrolidines, isoxazolidines

## Abstract

The reaction of *meso*-tetrakis(pentafluorophenyl)porpholactone with azomethine ylides and nitrones affords pyrrolidine-fused and isoxazolidine-fused dihydroporpholactones that display, respectively, isobacteriochlorin- and chlorin-type UV–Vis spectra. These reactions are site-selective, yielding, respectively, 17,18- or 12,13-dihydroporpholactones. The crystal and molecular features of pyrrolidine-fused and isoxazolidine-fused dihydroporpholactones were unveiled from single-crystal X-ray diffraction studies.

## 1. Introduction

Pyrrole-modified porphyrins have attracted considerable attention in recent years. This group of compounds comprises a large diversity of porphyrin analogues where one or more pyrrolic units of the porphyrin macrocycle are replaced by non-pyrrolic units. The synthesis, reactivity, structural features, optical properties, and applications of these compounds have been comprehensively reviewed [1,2,3,4,5,6]. In short, these compounds may be prepared by two complementary routes: by constructing the porphyrin macrocycle using adequate pyrrolic and non-pyrrolic building blocks or by chemical modification of already existing porphyrins. The latter route has been extensively used by the group of Brückner that, by using the “breaking and mending of porphyrins” approach [7], was able to produce a large diversity of pyrrole-modified porphyrins [2]. Focusing on their optical properties, these porphyrinoids may display porphyrin-, chlorin-, isobacteriochlorin-, or bacteriochlorin-like spectra, which is an important feature for a range of applications, including biological (photodynamic therapy (PDT) of cancer or multimodal imaging contrast agents) and technical ones (catalysis or optical sensors) [3,8,9,10,11,12].

Porpholactones, a class of pyrrole-modified porphyrins in which a pyrrolic unit is formally replaced by an oxazolone unit, can be conveniently obtained from *meso*-tetraarylporphyrins in a two-step procedure (osmium tetroxide-mediated dihydroxylation of the porphyrin followed by KMnO_4_-induced cleavage of 2,3-dihydroxychlorin) [13] or simply by oxidation of a *meso*-tetraarylporphyrin with Oxone^®^ in the presence of a catalytic amount of RuCl_3_ [14].

In the late 1990s, the group of Cavaleiro reported that the porphyrin macrocycle can participate as a dienophile in Diels–Alder reactions (D–A) [15] or as a dipolarophile in 1,3-dipolar cycloadditions (1,3-DC) [16]. The resulting monoadducts are heterocycle-fused chlorins, while bisadducts are bacteriochlorins or isobacteriochlorins. Concerning the 1,3-DC, the reaction of porphyrins with various types of 1,3-dipoles [17], namely, azomethine ylides [16,18,19] and nitrones [20,21], just to refer to the two types used in this work, has been reported. This is, effectively, an excellent and selective route to porphyrin derivatives with adequate optical properties for biological applications. Considering that 1,3-DC have also been successfully used for the peripheral functionalization of other porphyrin-type macrocycles, namely, tetraazaporphyrins [22], *N*-confused porphyrins [23], octaphyrins [24], and triply linked diporphyrins [25], the rarity of studies concerning the use of pyrrole-modified porphyrins as dipolarophiles is surprising [26]. In this article, we report the reaction of a porpholactone with two azomethine ylides and two nitrones. Since the porpholactone macrocycle is not symmetric, some site selectivity in these 1,3-DC reactions governed by the lactone unit was expected.

**Figure 1:**
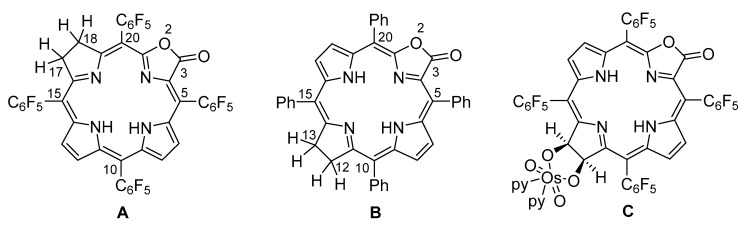
Known dihydroporpholactones structurally related to cycloadducts **3** and **6**/**7** [11,28,30].

Porpholactones display porphyrin-like UV–Vis spectra, which indicates that the lactone moiety mimics very well the electronic properties of a β–β’ bond [27]. However, the reactivity of the three remaining β–β’ bonds is influenced differently by the lactone unit. This influence is observed, for instance, when porpholactone **1** reacts with Woollins’ reagent and PhMe_2_SiH [28,29] to afford, selectively, a compound with a hydrogenated pyrrole unit adjacent to the lactone moiety (**A**, Figure 1) or when porpholactones react with hydrazine [30] (or tosylhydrazide) [31] to give compounds with a hydrogenated β–β’ bond opposite to the lactone moiety (**B**). The selective formation of *opp*-porphodilactones [32] and 12,13-dihydroxyporpholactone osmate esters (**C**) [11] from porpholactones are other examples of the orientation effect of the lactone unit. These observations prompted us to investigate the effect of the lactone unit on the reactivity of porpholactones with azomethine ylides and nitrones and, specifically, to find if it induces site selectivity and, eventually, regioselectivity. In these studies, we used *meso*-tetrakis(pentafluorophenyl)porpholactone (**1**) that was prepared by Zhang’s method [14].

## 2. Results and Discussion

**Figure 2:**
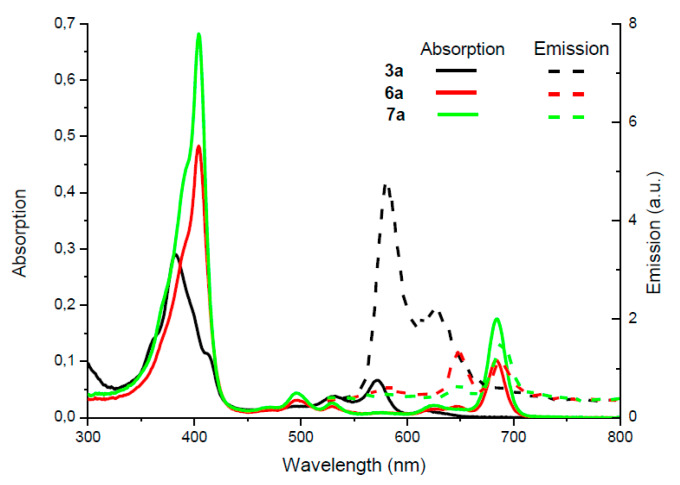
Absorption spectra of compounds **3a**, **6a**, and **7a** in chloroform (c = ~2 × 10^−6^ mol.L^−1^).

**Scheme 1:**
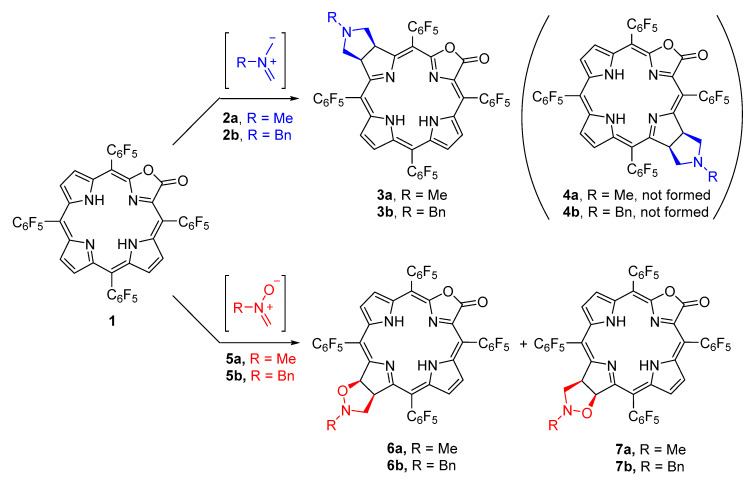
Reaction of porpholactone **1** with azomethine ylides **2a**, **b** and nitrones **5a**, **b**.

The reaction of porpholactone **1** with azomethine ylide **2a** (generated in situ from *N*-methylglycine and paraformaldehyde) was performed under the same reaction conditions applied to *meso*-tetrakis(pentafluorophenyl)porphyrin [16] (Scheme 1). Interestingly, a single monoadduct was formed, indicating clearly that the reaction was site-selective. A similar reaction with azomethine ylide **2b** (generated in situ from *N*-benzylglycine and paraformaldehyde) also afforded a single monoadduct. Both cycloadducts displayed isobacteriochlorin-type UV–Vis spectra (Figure 2 and Supplementary Materials) similar to that reported for dihydroporpholactone **A** [28], signifying that the cycloadditions occurred selectively at one of the pyrrolic units adjacent to the lactone moiety (i.e., they should have structures **3** or **4**). Their emission spectra were also similar to that of an isobacteriochlorin [28], with a main band around 580 nm and a shoulder around 630 nm. The quantum yields were determined to be 0.59 and 0.69 for **3a** and **3b**, respectively. These values also correlate well with the quantum yields reported for isobacteriochlorins. Their ^1^H NMR spectra revealed the signals of the NH protons at 3.58 and 4.50 ppm (for the *N*-methyl derivative) and at 3.58 and 4.60 ppm (for the *N*-benzyl derivative). These signals disappeared after shaking with D_2_O. ^1^H NMR spectra of porphyrin derivatives with NH signals at such low fields are typical of isobacteriochlorins [15,18,21,33,34], and the same pattern is also observed in isobacteriochlorin-type structures such as compound **A** [28].

**Figure 3:**
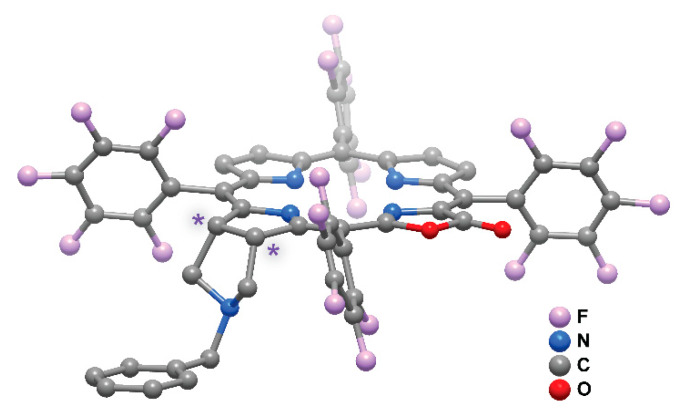
Ball-and-stick representation of the pyrrolidine-fused porpholactone derivative **3b**. The chiral centers are indicated by an asterisk. Hydrogen atoms were omitted for clarity reasons.

The *N*-benzyl cycloadduct led to the isolation of poor crystals, which could be, nevertheless, investigated using single-crystal X-ray diffraction studies. The crystallographic studies performed revealed that the material crystallized in the centrosymmetric *P*-1 triclinic space group, with the asymmetric unit being composed of two disordered halves of two independent molecular units. A full disclosure of the structure of the cycloadduct was instead achieved by solving and refining the structure in the *P*1 triclinic space group (by discarding the center of inversion). With this procedure, it was possible to unequivocally confirm that the cycloadduct has structure **3b** (and not **4b**, see Scheme 1 and Figure 3). Though the X-ray diffraction reliability factors were high (due to the poor diffraction of the crystal), the disclosed structural features were in perfect agreement with those obtained from additional characterization techniques, namely, NMR and UV–Vis spectroscopy.

**Figure 4:**
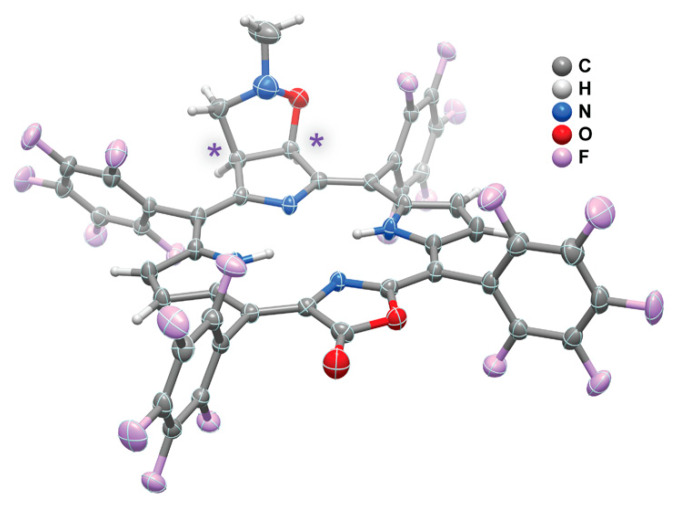
Oak Ridge Thermal Ellipsoid Plot (ORTEP) representation of the nitrone cycloadduct **6a** derivative. Non-hydrogen atoms are represented as thermal ellipsoids drawn at the 50% probability level, and hydrogen atoms as small spheres with arbitrary radii. The chiral centers are indicated by an asterisk.

The reaction of porpholactone **1** with nitrone **5a** (generated in situ from *N*-methylhydroxylamine hydrochloride and paraformaldehyde in the presence of potassium carbonate) was performed under the same reaction conditions applied to *meso*-tetrakis(pentafluorophenyl)porphyrin [21]. TLC analysis of the reaction mixture showed one spot corresponding to porpholactone **1** and two very close spots corresponding to new compounds. The unreacted porpholactone and the two products could be separated by preparative TLC (silica gel) using a 1:1 mixture of CH_2_Cl_2_/hexane as the eluent. Similarly, when nitrone **5b** was used (generated in situ from *N*-benzylhydroxylamine hydrochloride and paraformaldehyde in the presence of potassium carbonate), two products with similar R_f_ were also obtained. The UV–Vis spectra of the new compounds were identical to that of 12,13-dihydroxyporpholactone osmate ester **C**,^11^ showing Q bands at λ_max_ 682 nm (Figure 2). These spectra clearly indicate that the cycloadditions occurred selectively at the pyrrolic unit opposite to the lactone moiety. Consequently, the cycloadducts are regioisomers **6** and **7** [35]. The mass spectra of these products indicated that they are all monoadducts, showing the protonated molecular peaks (M + H)^+^ = 1052 for **6a** and **7a** and (M + H)^+^ = 1128 for **6b** and **7b**. Each pair of regioisomers was obtained in a ratio of ca. 2.4:1, indicating that cycloaddition, besides being site-selective, was also regioselective. A slow crystallization of the main cycloadduct formed in the reaction with nitrone **5a** allowed obtaining crystals adequate for single-crystal X-ray diffraction studies. These studies revealed that it has structure **6a** (Figure 4) and, therefore, the minor isomer has structure **7a**.

The emission spectra of adducts **6** and **7** presented two bands around 650 nm and 690 nm and quantum yields between 0.14 and 0.23, which were much smaller than those for adducts **3** (Table S1, SM). The ^1^H NMR spectra of compounds **6** and **7** were markedly different from those of compounds **3a**/**3b**, particularly at the chemical shifts of the signals of the NH protons: −1.53 and −1.19 ppm for **6a**, −1.67 and −1.36 ppm for **7a**, −1.68 and −1.31 ppm for **6b**, and −1.73 and −1.43 ppm for **7b**. The ^13^C and ^19^F NMR spectra of the cycloadducts discussed above are also compatible with the proposed structures, displaying various sets of peaks as expected for porpholactone derivatives with reduced symmetry. In contrast to azomethine adducts **3**, nitrone cycloadducts **6** and **7** revealed to be unstable in solution at room temperature, affording, slowly, porpholactone **1**. This transformation, probably via retrocycloaddition, was easily observed by TLC and was also detected in the ^13^C NMR spectra of the cycloadducts that showed a signal corresponding to the carbonyl group of the adduct and a small one due to the carbonyl group of porpholactone **1**.

In a previous work [21], we have shown that the addition of an azomethine ylide to a chlorin results in the formation of an isobacteriochlorin, while the addition of a nitrone to a chlorin leads to the formation of a bacteriochlorin. Although being electronically related to porphyrins (and not to chlorins), porpholactones react with azomethine ylides and nitrones with site selectivity identical to that expected for a chlorin, and this is a noteworthy observation. The reason for the site selectivity observed in the reaction of 1,3-DC with chlorins was elucidated by a DFT study [36]: while the addition of an azomethine ylide is irreversible and kinetically controlled, the addition of a nitrone is reversible, pointing to a thermodynamic control of the reaction. Experimental observations in this work, namely, the retrocycloaddition observed with nitrone cycloadducts, suggest that a similar reaction control also operates with porpholactones.

In conclusion, this study showed that the reactions of *meso*-tetrakis(pentafluorophenyl)porpholactone with 1,3-dipoles are site-selective and, depending on the type of 1,3-dipole used, the resulting cycloadducts are 12,13- or 17,18-dihydroporpholactones, which display, respectively, chlorin- or isobacteriochlorin-type UV–Vis spectra. Considering that a large diversity of substituents may be linked to these cycloadducts by nucleophilic substitutions at the pentafluorophenyl groups [37,38], the reaction of porpholactone **1** with 1,3-dipoles may be an excellent route to a myriad of compounds for biological or technical applications.

## 3. Materials and Methods

### 3.1. Chemicals and Instrumentation

^1^H, ^13^C and ^19^F NMR spectra were recorded on Bruker AVANCE 300 or Bruker AVANCE 500 spectrometers. CDCl_3_ was used as solvent, and tetramethylsilane (TMS) as an internal reference. The chemical shifts are expressed in δ (ppm), and the coupling constants (*J*) in hertz (Hz). UV–Vis spectra were recorded on a Shimadzu UV-2501PC spectrophotometer using CHCl_3_ as the solvent. Fluorescence emission spectra were recorded with a Jobin Yvon FluoroMax-3 spectrofluorometer using CHCl_3_ as the solvent. Fluorescence quantum yields Φ_F_ were determined using 5,10,15,20-tetraphenylporphyrin (TPP) in chloroform (Φ_F_ = 0.11) as the fluorescence standard. Mass spectra were recorded using a Micromass Q-TOF-2^TM^ mass spectrometer and CHCl_3_ as the solvent. Preparative thin-layer chromatography was carried out on 20 × 20 cm glass plates coated with silica gel (1 mm thick, Merck, Kenilworth, NJ, USA). Analytical TLC was carried out on sheets precoated with silica gel (Merck 60, 0.2 mm thick). Solvents were purified or dried according to the literature procedures. Compound **1** was prepared according to a published procedure [14].

### 3.2. Synthesis

#### 3.2.1. General Procedure for the Synthesis of Cycloadducts **3**.

A solution of porpholactone **1** (50.0 mg, 0.05 mmol), N-substituted glycine (0.45 mmol), and paraformaldehyde (33.8 mg, 1.1 mmol) in toluene (25 mL) was heated at reflux under a nitrogen atmosphere for 4 h. Then, new portions of the N-substituted glycine (0.45 mmol) and paraformaldehyde (33.8 mg, 1.1 mmol) were added, and the mixture was refluxed for additional 4 h. The solvent was evaporated, and the compounds were separated by preparative TLC using dichloromethane/hexane as the eluent. The compound with higher R_f_ corresponded to unreacted porpholactone (~10%), and the fraction running immediately below it corresponded to the expected cycloadduct. Isolated yields: **3a** = 85%; **3b** = 43%.

Adduct **3a**: (45.2 mg, 85% yield), ^1^H NMR (300 MHz, CDCl_3_): δ 2.23 (s, 3H, CH_3_), 2.44–2.54 (m, 2H, pyrrolidine-*H*), 2.84–2.98 (m, 2H, pyrrolidine-*H*), 3.58 (s, 1H, NH), 4.48–4.71 (m, 3H, NH and β-H [C (sp^3^)]), 7.32 (dd, 1H, *J* = 4.8 and 1.4 Hz, β-H), 7.59 (d, 2H, *J* = 1.4 Hz, β-H), 7.77 (d, 1H, *J* = 4.8 Hz, β-H). ^13^C NMR (75 MHz, CDCl_3_): δ 40.9, 47.6, 48.8, 62.0, 62.2, 83.2, 95.7, 109.3, 110.2, 110.6, 112.5, 113.7, 114.1, 122.0, 123.3, 134.7, 135.6, 135.9, 137.0, 138.5, 139.0, 141.2, 143.2, 144.1, 144.8, 145.4, 146.1, 146.6, 147.3, 151.2, 154.1, 157.6, 159.1, 162.2, 164.6. ^19^F NMR (282 MHz, CDCl_3_): δ −158.0 to −155.9 (m, 8H, *m*-F), −148.3 to −147.2 (m, 4H, *p*-F), −135.6 to −132.0 (m, 8H, *o*-F). UV-Vis (CHCl_3_): λ_max_ (log ε) 382 (5.23), 531 (4.38), 572 (4.60), 617 (3.93). MS (ESI): *m*/*z* 1050.5 [M + H]^+^.

Adduct **3b**: (24.2 mg, 43% yield), ^1^H NMR (300 MHz, CDCl_3_): δ 2.53–2.60 (m, 2H, pyrrolidine-*H*), 2.84 (t, *J* = 8.7 Hz, 2H, pyrrolidine-*H*), 3.46 (s, 2H, PhCH_2_), 3.58 (s, 1H, NH), 4.45–4.63 (m, 3H, NH and β-H [C (sp^3^)]), 7.06–7.09 (m, 2H, Bn-H), 7.15–7.18 (m, 1H, Bn-H), 7.28–7.30 (m, 3H, Bn-H and β-H), 7.59 (d, 2H, *J* = 1.6 Hz, β-H), 7.77 (d, 1H, *J* = 4.8 Hz, β-H). ^13^C NMR (125 MHz, CDCl_3_): δ 47.0, 48.2, 59.1, 59.7, 59.9, 83.1, 95.7, 109.4, 110.2, 110.5, 112.5, 114.0, 122.0, 123.2, 125.3, 127.7, 128.2, 128.4, 128.8, 129.05, 129.11, 134.8, 135.6, 135.8, 136.8, 138.8, 141.0, 143.2, 144.0, 144.5, 145.2, 146.0, 146.4, 146.8, 147.3, 151.3, 154.1, 157.6, 159.1, 162.3, 164.6. ^19^F NMR (282 MHz, CDCl_3_): δ −158.0 to −155.9 (m, 8H, *m*-F), −148.3 to −147.3 (m, 4H, *p*-F), −135.7 to −131.8 (m, 8H, *o*-F). UV-Vis (CHCl_3_): λ_max_ (log ε) 382 (5.21), 532 (4.34), 572 (4.59), 629 (3.56). MS (ESI): *m*/*z* 1126.1 [M + H]^+^.

#### 3.2.2. General Procedure for the Synthesis of Cycloadducts **6** and **7**.

To a solution of porpholactone **1** (50.0 mg, 0.05 mmol) in toluene (5 mL), *N*-substituted hydroxylamine hydrochloride (0.20 mmol), paraformaldehyde (15.1 mg, 0.50 mmol), and K_2_CO_3_ (55.7 mg, 0.40 mmol) were added. The mixture was heated at 60 °C under a nitrogen atmosphere for 12 h, and then additional portions of *N*-substituted hydroxylamine hydrochloride, paraformaldehyde, and K_2_CO_3_ were added to the reaction mixture, which was heated for a total of 30 h. The reaction mixture was washed with H_2_O and then was purified by preparative TLC using dichloromethane/hexane (1:1) as the eluent. The fraction with higher R_f_ corresponded to unreacted porpholactone (~60%), the following one was the major cycloadduct, and the third one the minor cycloadduct. Isolated yields: **6a** = 19% (48% yield based on consumed porpholactone **1**), **7a** = 8% (19% yield based on consumed porpholactone **1**); **6b** = 22% (43% yield based on consumed porpholactone **1**), **7b** = 9% (18% yield based on consumed porpholactone **1**).

Adduct **6a**: ^1^H NMR (300 MHz, CDCl_3_): δ −1.53 (s, 1H, NH), −1.19 (s, 1H, NH), 2.57 (s, 3H, CH_3_), 2.86–2.95 (m, 1H, isoxazolidine-H), 3.35–3.43 (m, 1H, isoxazolidine-H), 5.14–5.19 (m, 1H, β-H [C (sp^3^)]), 6.65 (d, 1H, *J* = 6.8 Hz, β-H [C (sp^3^)]), 8.19 (s, 1H, β-H), 8.48–8.52 (m, 2H, β-H), 8.64 (d, 1H, *J* = 4.0 Hz, β-H). ^13^C NMR (75 MHz, CDCl_3_): δ 44.0, 55.4, 64.0, 85.0, 89.7, 98.7, 102.3; 105.5, 105.8, 110.7, 111.4, 114.5; 122.9, 125.3, 126.8, 127.9, 128.1, 128.2, 128.3, 134.7, 137.5, 138.2, 138.9, 140.9, 141.6, 143.5, 145.1, 147.1, 148.9, 152.6, 162.9, 165.8. ^19^F NMR (282 MHz, CDCl_3_): δ −158.2 to −155.7 (m, 8H, *m*-F), −148.3 to −146.7 (m, 4H, *p*-F), −136.0 to −131.1 (m, 8H, *o*-F). UV-vis (CHCl_3_): λ_max_ (log ε) 404 (5.30), 497 (4.07), 530 (3.86), 625 (3.75), 684 (4.61). MS (ESI): *m*/*z* 1052.5 [M + H]^+^.

Adduct **7a**: ^1^H NMR (300 MHz, CDCl_3_): δ −1.67 (s, 1H, NH), −1.36 (s, 1H, NH), 2.57 (s, 3H, CH_3_), 2.90–2.95 (m, 1H, isoxazolidine-H), 3.39–3.44 (m, 1H, isoxazolidine-H), 5.21–5.23 (m, 1H, β-H [C (sp^3^)]), 6.66 (d, 1H, *J* = 7.4 Hz, β-H [C (sp^3^)]), 8.33 (d, 1H, *J* = 4.5 Hz, β-H), 8.40 (d, 1H, *J* = 4.4 Hz, β-H), 8.56 (d, 1H, *J* = 4.4 Hz, β-H), 8.66 (d, 1H, *J* = 4.5 Hz, β-H). ^19^F NMR (282 MHz, CDCl_3_): δ −158.4 to −155.6 (m, 8H, *m*-F), −148.7 to −146.1 (m, 4H, *p*-F), −136.3 to −131.2 (m, 8H, *o*-F). UV-vis (CHCl_3_): λ_max_ (log ε) 404 (5.44), 496 (4.20), 530 (3.97), 625 (3.90), 684 (4.83). MS (ESI): *m*/*z* 1052.2 [M + H]^+^.

Adduct **6b**: ^1^H NMR (300 MHz, CDCl_3_): δ −1.68 (s, 1H, NH), −1.31 (s, 1H, NH), 3.25 (s, 2H, isoxazolidine-*H*), 3.81 (s, 2H, PhCH_2_), 5.24–5.25 (m, 1H, isoxazolidine-*H*), 6.65 (d, 1H, *J* = 7.4 Hz, β-H [C (sp^3^)]), 7.00–7.02 (m, 2H, Bn-H), 7.19–7.22 (m, 3H, Bn-H), 8.22 (d, 1H, *J* = 3.2 Hz, β-H), 8.46 (d, 1H, *J* = 4.1 Hz, β-H), 8.55 (d, 1H, *J* = 4.1 Hz, β-H), 8.66 (d, 1H, *J* = 3.2 Hz, β-H). ^13^C NMR (125 MHz, CDCl_3_): δ 44.0, 53.9, 64.6, 86.1, 89.3, 99.6, 101.0, 105.8, 110.7, 112.7, 113.7, 115.3, 122.3, 125.4, 127.3, 128.3, 136.8, 137.5, 138.9, 139.3, 139.4, 141.2, 141.6, 143.3, 143.7, 144.8, 145.2, 146.9, 152.9, 163.0, 165.8. ^19^F NMR (282 MHz, CDCl_3_): δ −158.2 to −155.8 (m, 8H, *m*-F), −148.2 to −146.7 (m, 4H, *p*-F), −135.7 to −130.1 (m, 8H, *o*-F). UV-vis (CHCl_3_): λ_max_ (log ε) 404 (5.92), 496 (4.66), 529 (4.46), 623 (4.35), 646 (4.33), 682 (5.24). MS (ESI): *m*/*z* 1128.4 [M + H]^+^.

Adduct **7b**: ^1^H NMR (300 MHz, CDCl_3_): δ −1.73 (s, 1H, NH), −1.43 (s, 1H, NH), 3.31 (s, 2H, isoxazolidine-*H*), 3.85 (s, 2H, PhCH_2_), 5.33–5.37 (m, 1H, isoxazolidine-*H*), 6.67 (d, 1H, *J* = 7.8 Hz, β-H [C (sp^3^)]), 7.02–7.06 (m, 2H, Bn-H), 7.19–7.23 (m, 3H, Bn-H), 8.30 (d, 1H, *J* = 5.10 Hz, β-H), 8.42 (d, 1H, *J* = 4.2 Hz, β-H), 8.57 (d, 1H, *J* = 4.2 Hz, β-H), 8.68 (d, 1H, *J* = 5.1 Hz, β-H). ^19^F NMR (282 MHz, CDCl_3_): δ −158.3 to 155.5 (m, 8H, *m*-F), −148.3 to −146.1 (m, 4H, *p*-F), −136.3 to −130.0 (m, 8H, *o*-F). UV-vis (CHCl_3_): λ_max_ (log ε) 404 (5.57), 497 (4.30), 529 (4.06), 626 (3.97), 643 (4.05), 684 (4.87). MS (ESI): *m*/*z* 1128.3 [M + H]^+^.

CCDC 1832879, 1832880, and 1985131 contain the supplementary crystallographic data for this paper. These data can be obtained free of charge via www.ccdc.cam.ac.uk/data_request/cif, by emailing data_request@ccdc.cam.ac.uk, or by contacting The Cambridge Crystallographic Data Centre, 12 Union Road, Cambridge CB2 1EZ, UK; fax: +44 1223 336033.

## Supplementary Materials

The following are available online. ^1^H, ^13^C and ^19^F NMR spectra, mass spectra, absorption, and emission spectra for the new compounds. Details of the crystal data collection, solution, and refinement of compounds **3b** and **6a**. Crystal structures in CIF format (CIF).
