# Supplementary material for: Site-Selective Modification of a Porpholactone—Selective Synthesis of 12,13- and 17,18-Dihydroporpholactones
Source: Molecules. 2020 Jun 6;25(11):2642. doi: 10.3390/molecules25112642 (PMC7321334; doi:10.3390/molecules25112642)
Supplement: Supplementary file 1 [file molecules-25-02642-s001.pdf]

Supplementary material

Site selective modification of a porpholactone – selective synthesis of 12,13- and 17,18-dihydroporpholactones

Ana F.R. Cerqueira,[a] Gustautas Snarskis,[a] Jonas Zurauskas,[a] Samuel Guieu,[a,b] Filipe A. Almeida Paz,[b] and Augusto C. Tomé[a,]*

[a] LAQV-REQUIMTE, Department of Chemistry, University of Aveiro, 3810-193 Aveiro, Portugal

[b] CICECO – Aveiro Institute of Materials, Department of Chemistry, University of Aveiro, 3810-193 Aveiro, Portugal

Contents

NMR, Mass and UV-Vis spectra...2

Photophysical studies 16

HPLC analysis...17

Single crystal X-ray diffraction studies 17

NMR, Mass and UV-Vis spectra

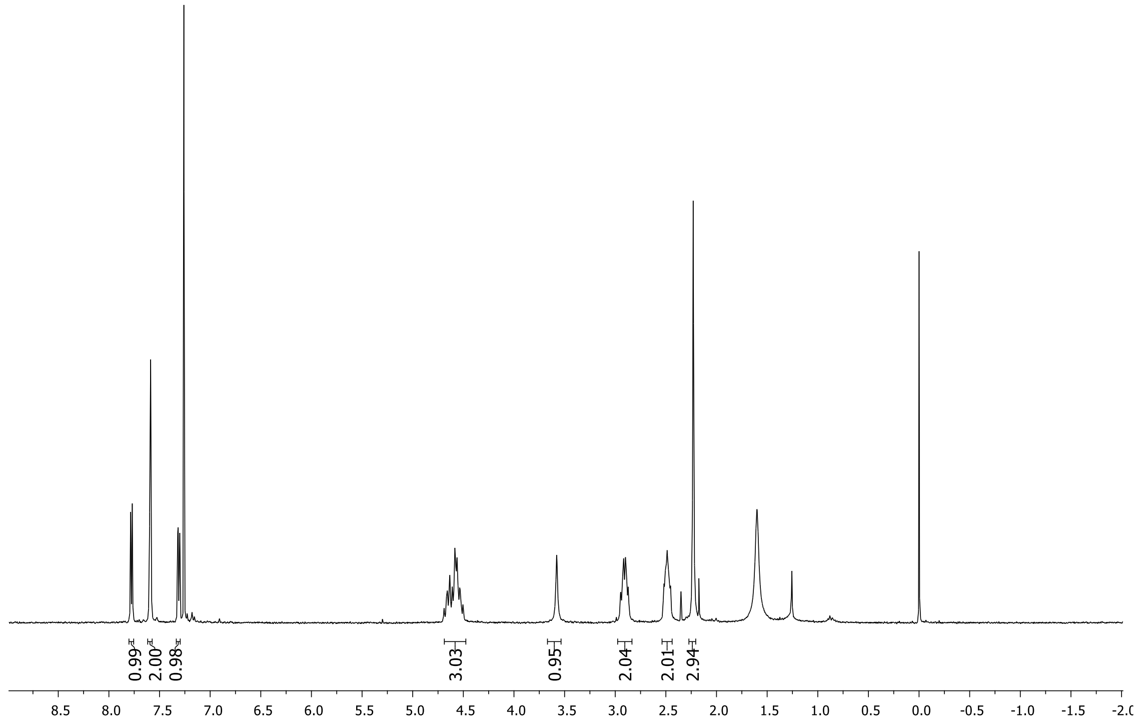

Fig. 1. ^{1}H NMR spectrum of compound **3a** (300 MHz, $CDCl_3$).

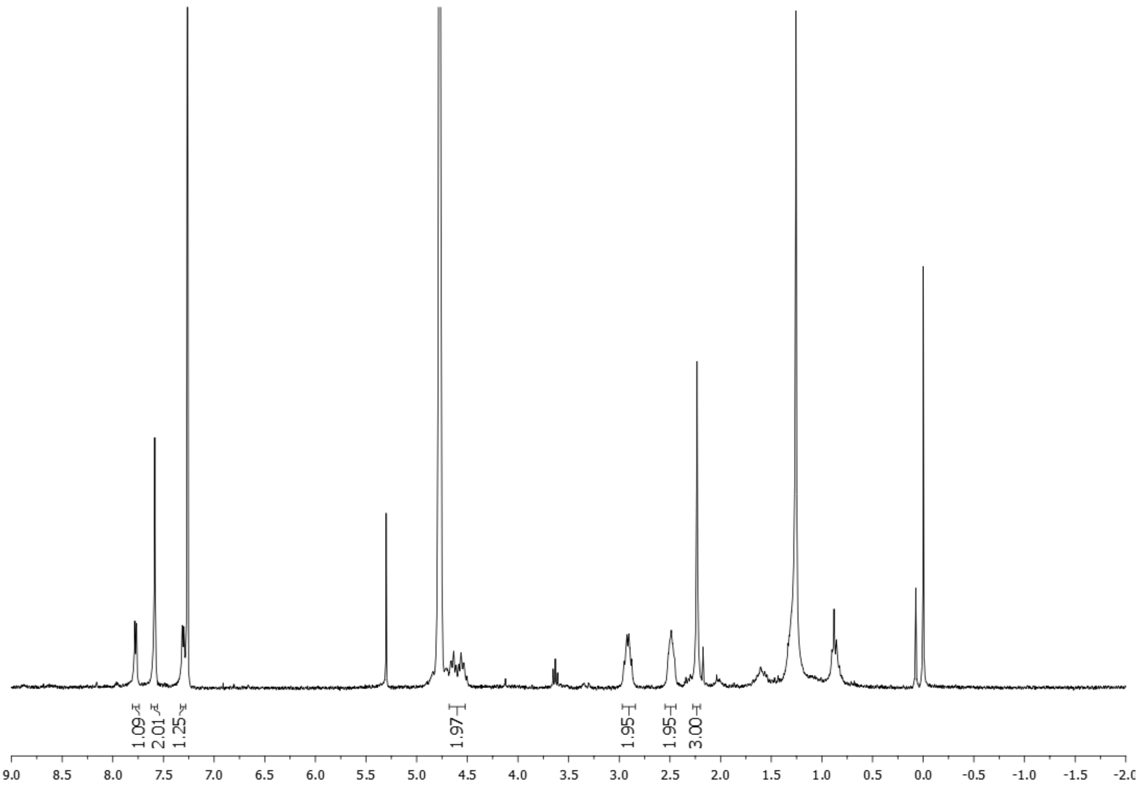

Fig. 2. ^{1}H NMR spectrum of compound **3a** (300 MHz, $CDCl_3$ with addition of a few drops of D_2O).

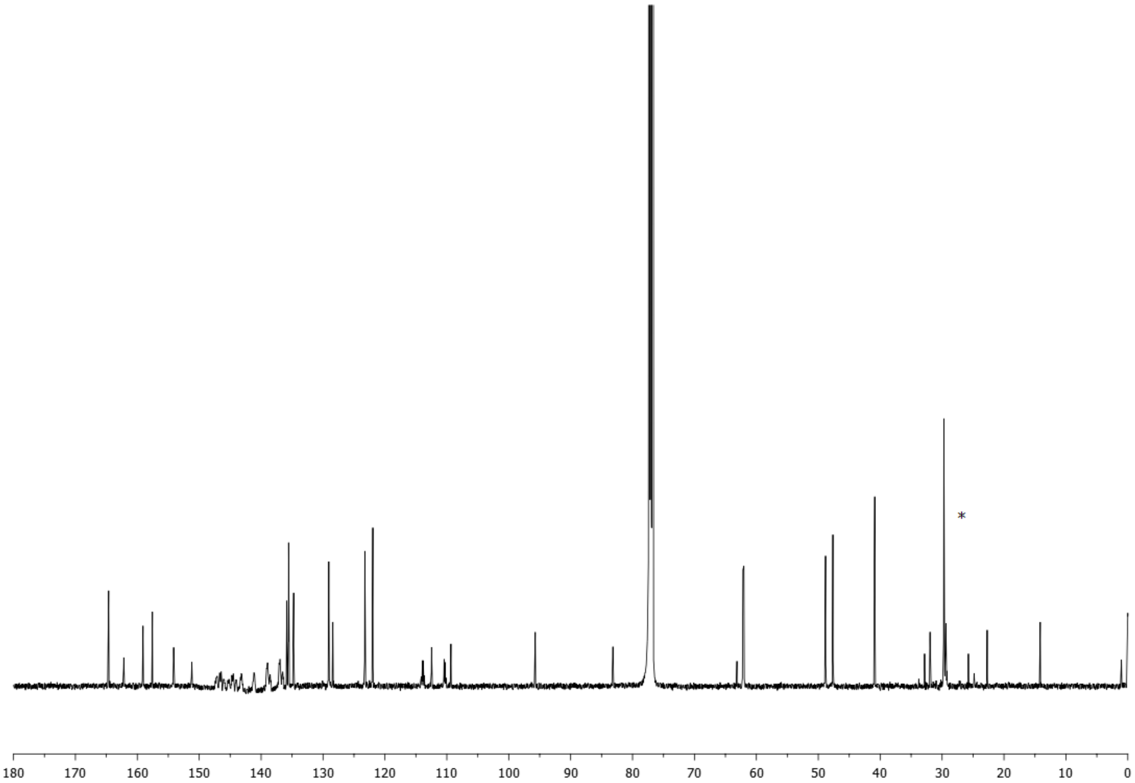

Fig. 3. ^{13}C NMR spectrum of compound **3a** (75 MHz, $CDCl_3$).

* Solvents used in the crystallization.

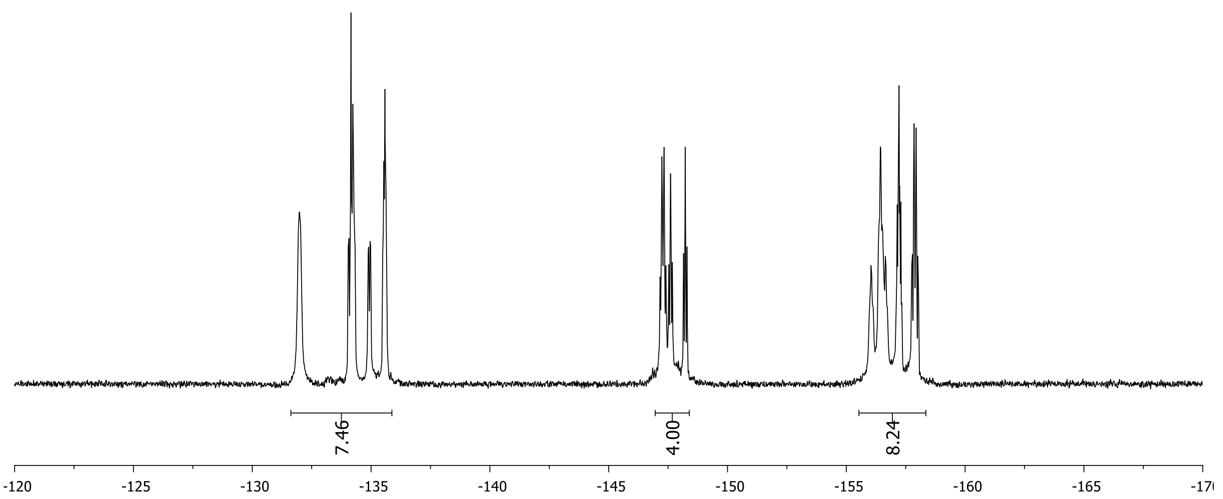

Fig. 4. ^{19}F NMR spectrum of compound **3a** (282 MHz, $CDCl_3$).

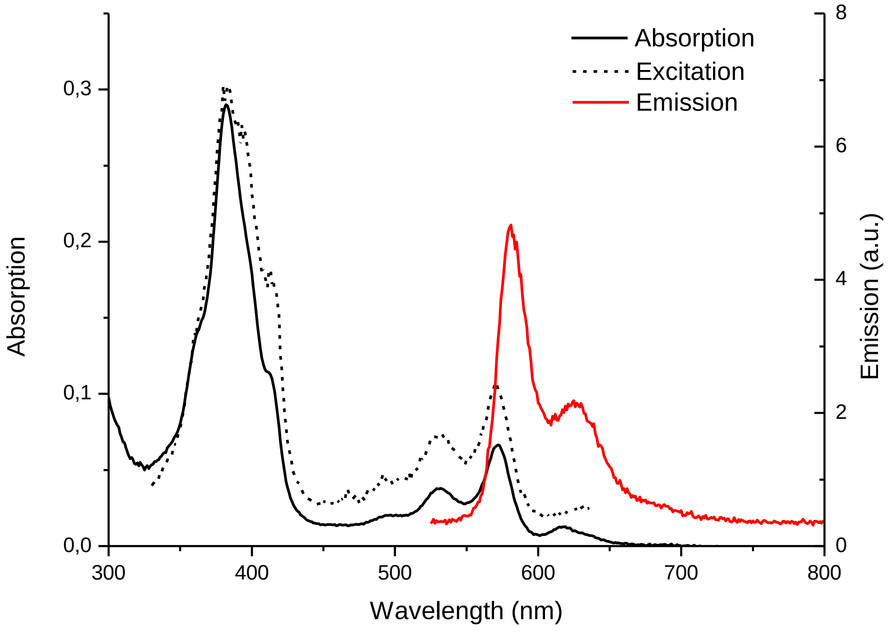

Fig. 5. Absorption, excitation (λ_{em} = 650 nm) and emission spectra (λ_{ex} = 515 nm) of compound **3a** ($1.6x10^{-6}$ $mol.L^{-1}$ in $CHCl_3$).

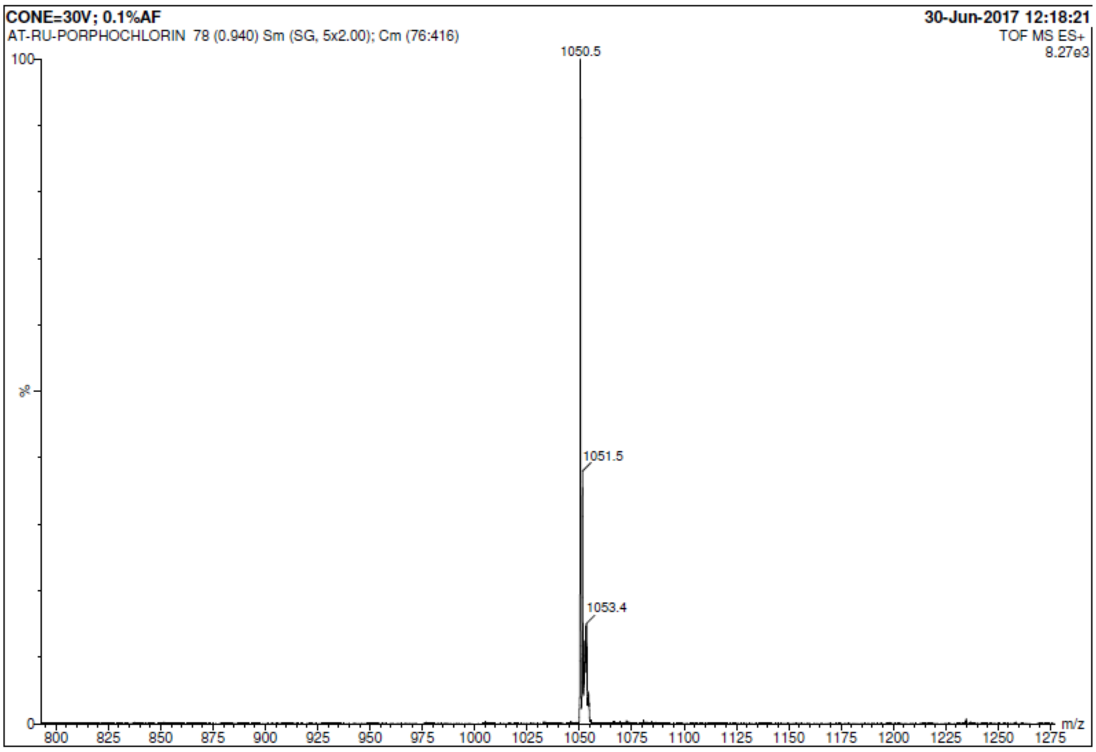

Fig. 6. Mass spectrum of compound **3a**.

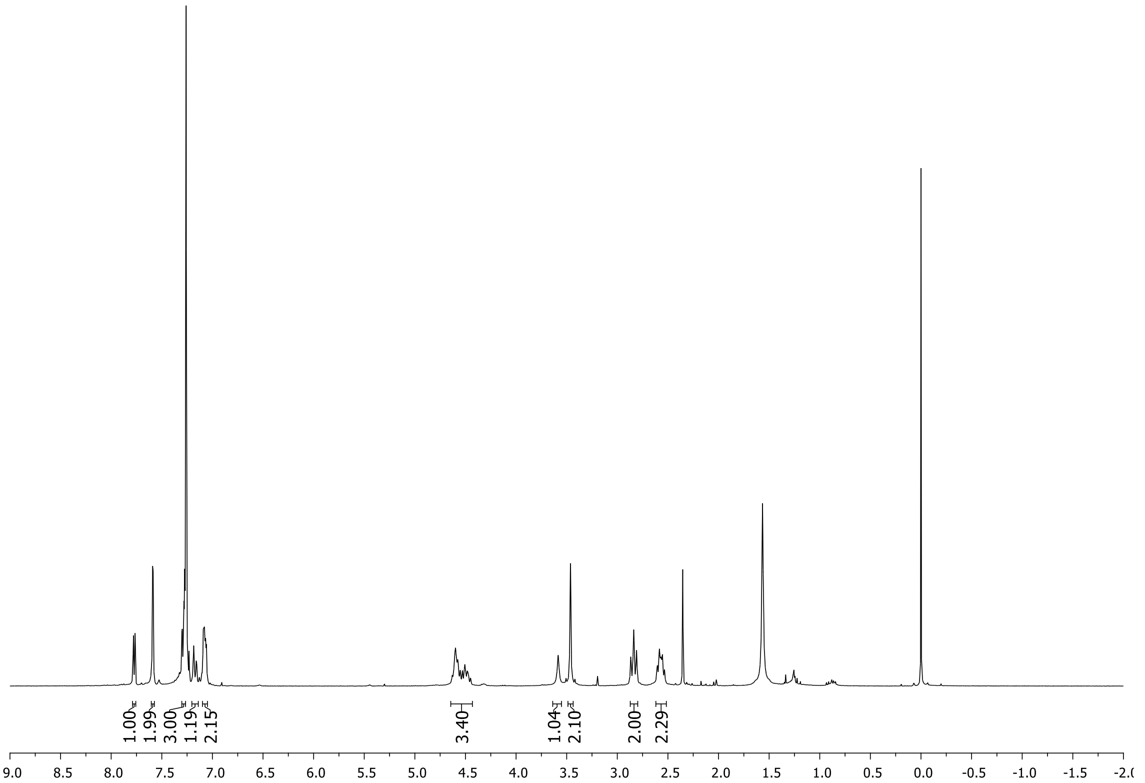

Fig. 7. ^{1}H NMR spectrum of compound **3b** (300 MHz, $CDCl_3$).

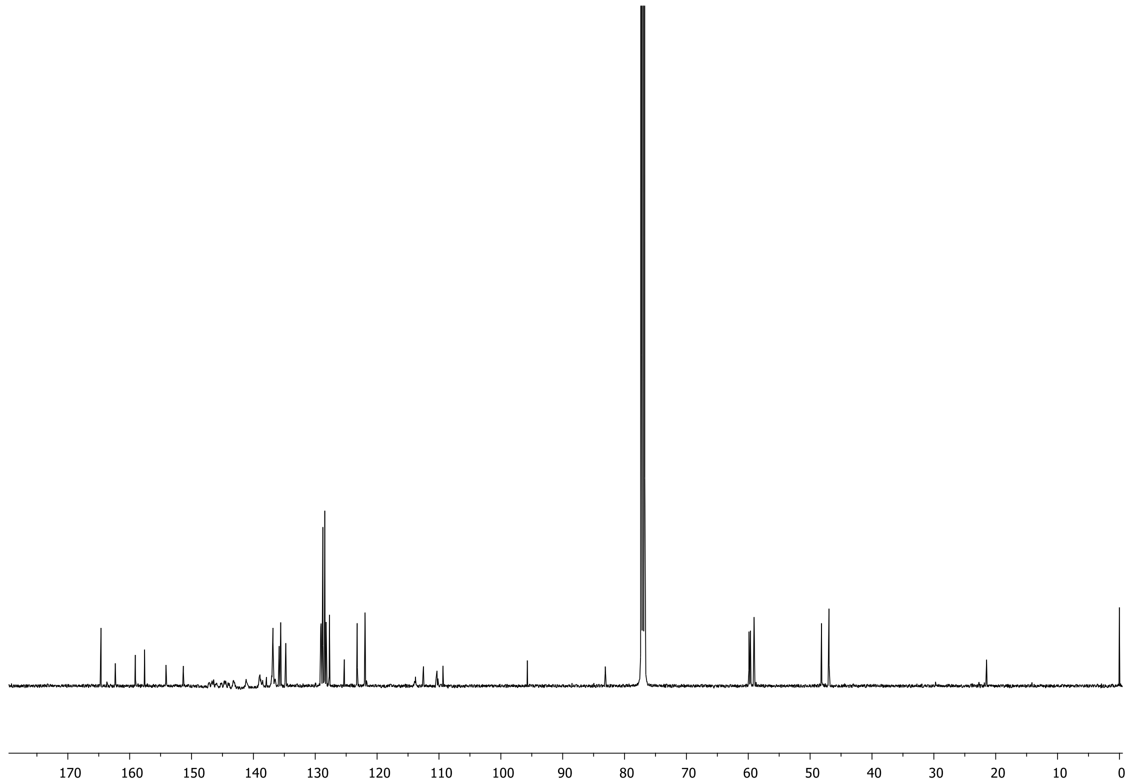

Fig. 8. ^{13}C NMR spectrum of compound **3b** (125 MHz, $CDCl_3$).

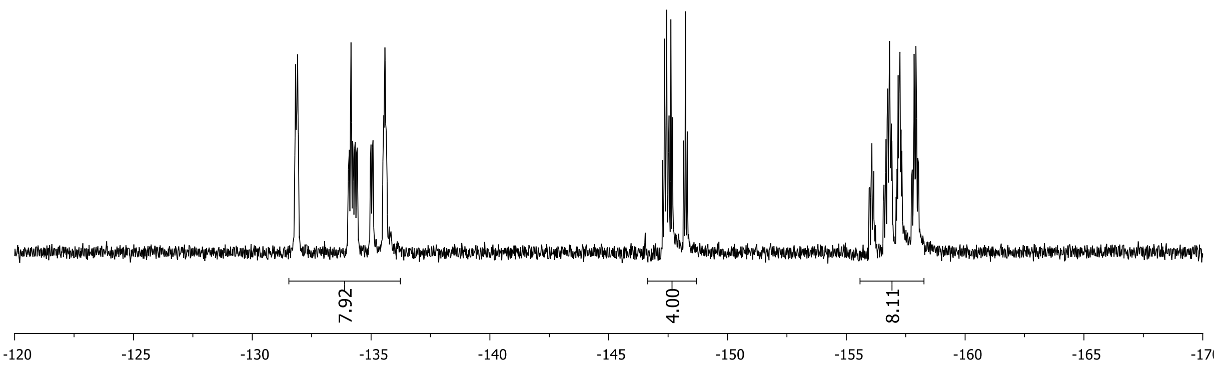

Fig. 9. ^{19}F NMR spectrum of compound **3b** (282 MHz, $CDCl_3$).

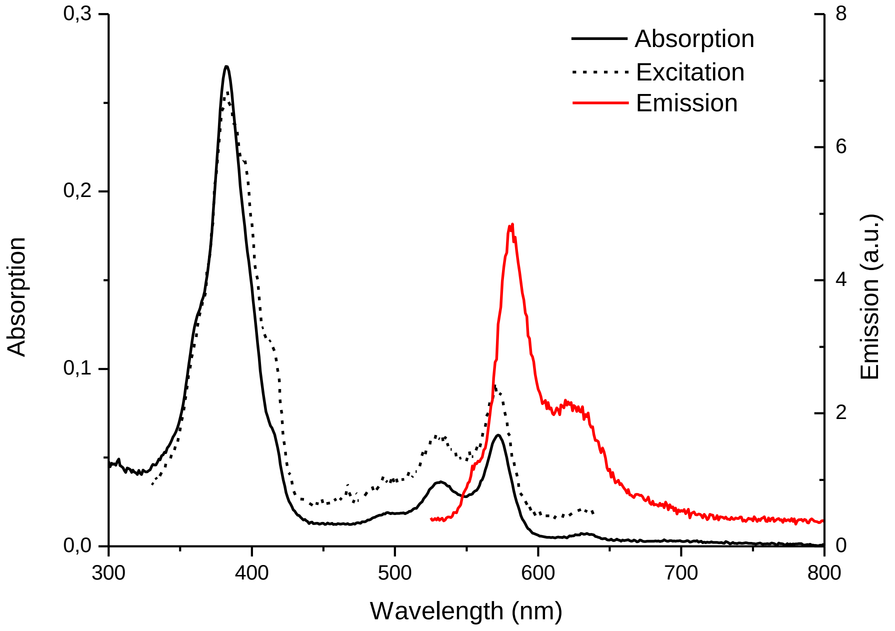

Fig. 10. Absorption, excitation (λ_{em} = 650 nm) and emission spectra (λ_{ex} = 515 nm) of compound **3b** ($1.5x10^{-6}$ $mol.L^{-1}$ in $CHCl_3$).

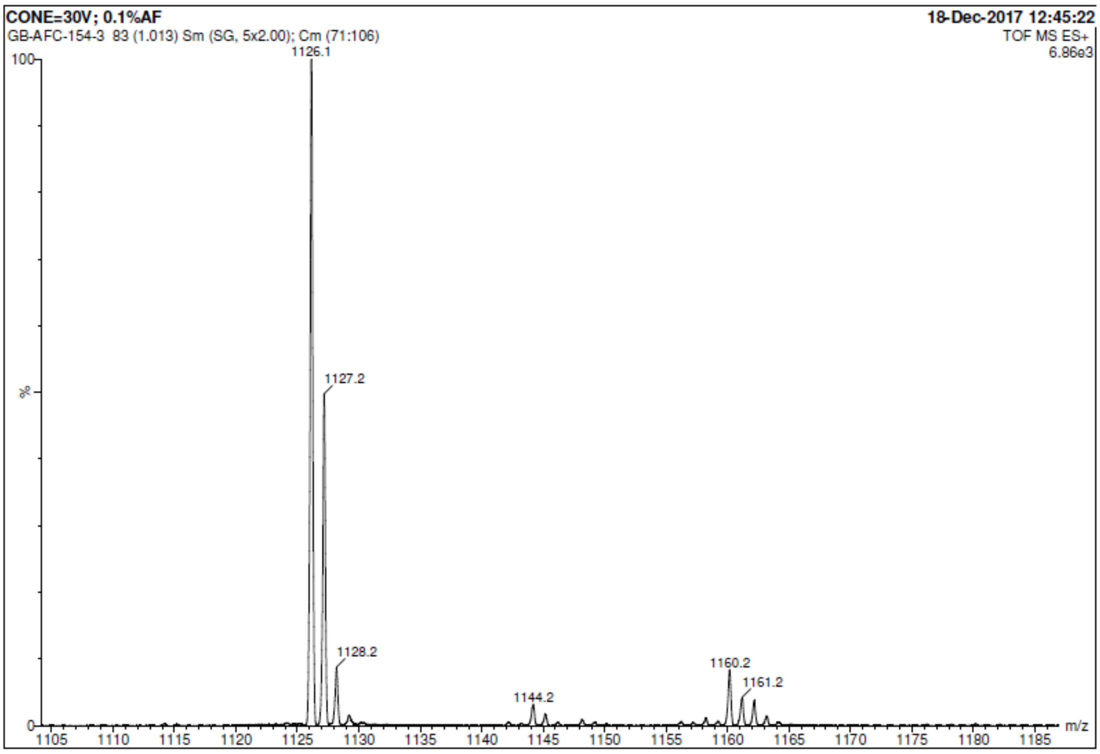

Fig. 11. Mass spectrum of compound **3b**.

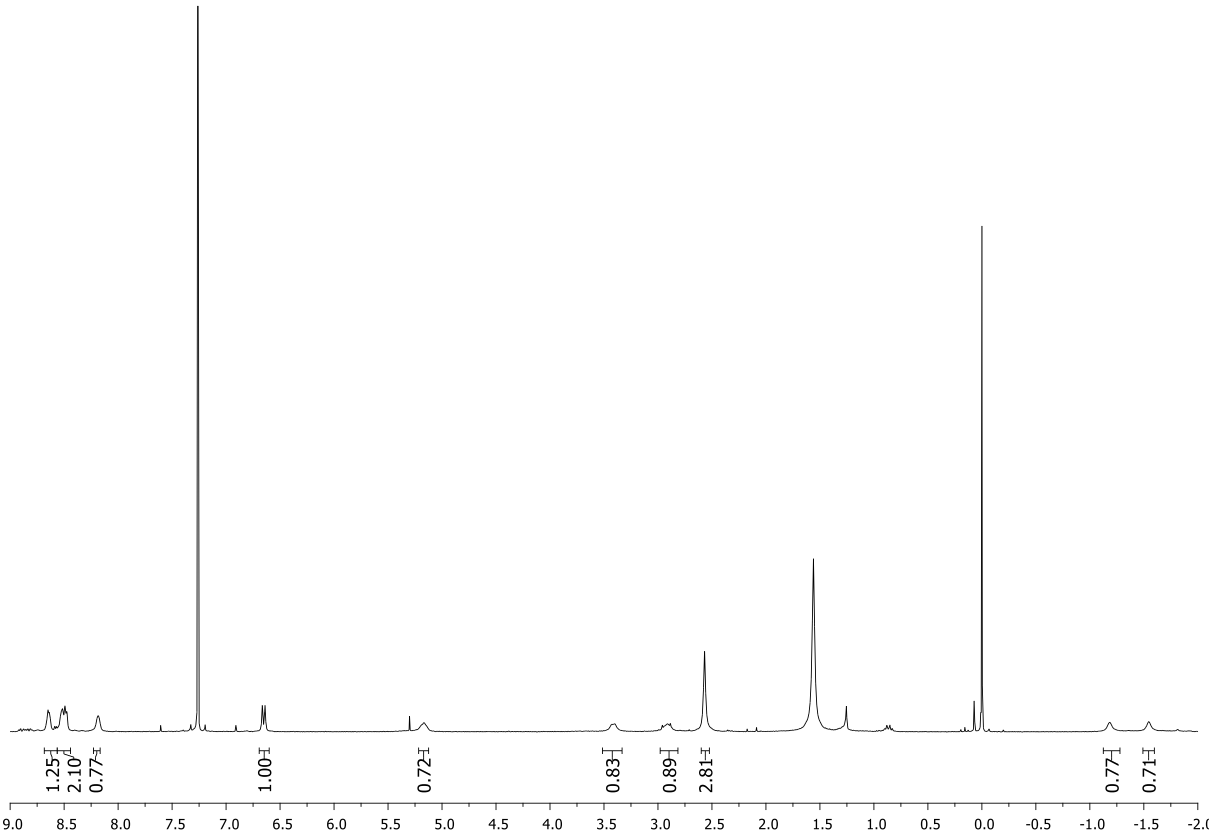

Fig. 12. ^{1}H NMR spectrum of the nitrone cycloadduct **6a** (300 MHz, $CDCl_3$).

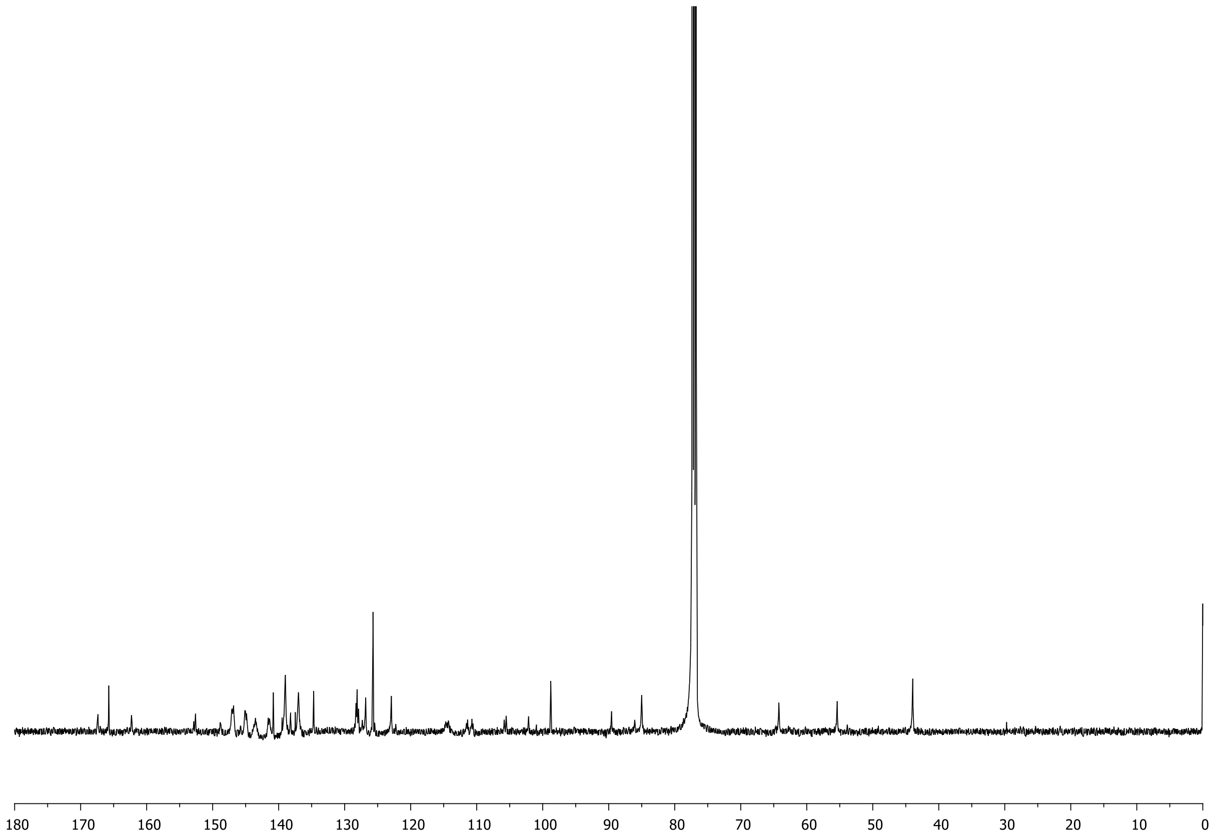

Fig. 13. ^{13}C NMR spectrum of the nitrone cycloadduct **6a** (125 MHz, $CDCl_3$).

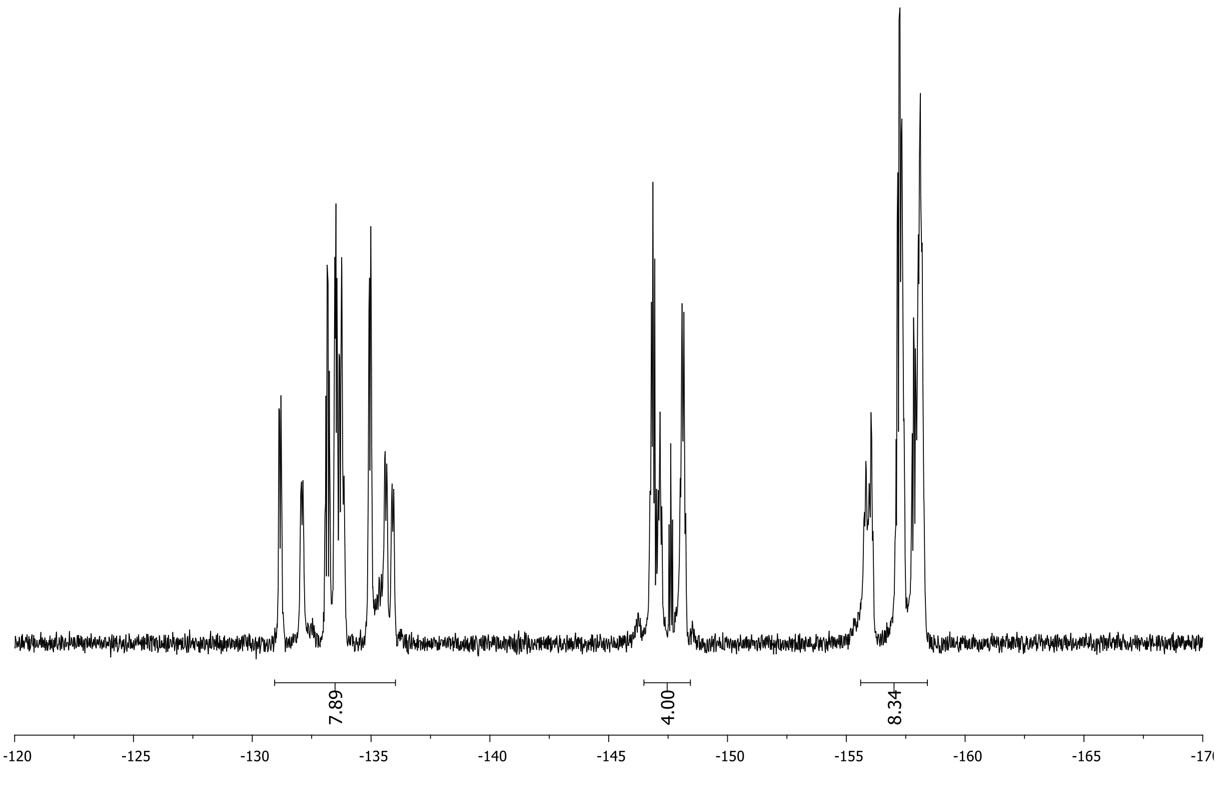

Fig. 14. ^{19}F NMR spectrum of the nitrone cycloadduct **6a** (282 MHz, $CDCl_3$).

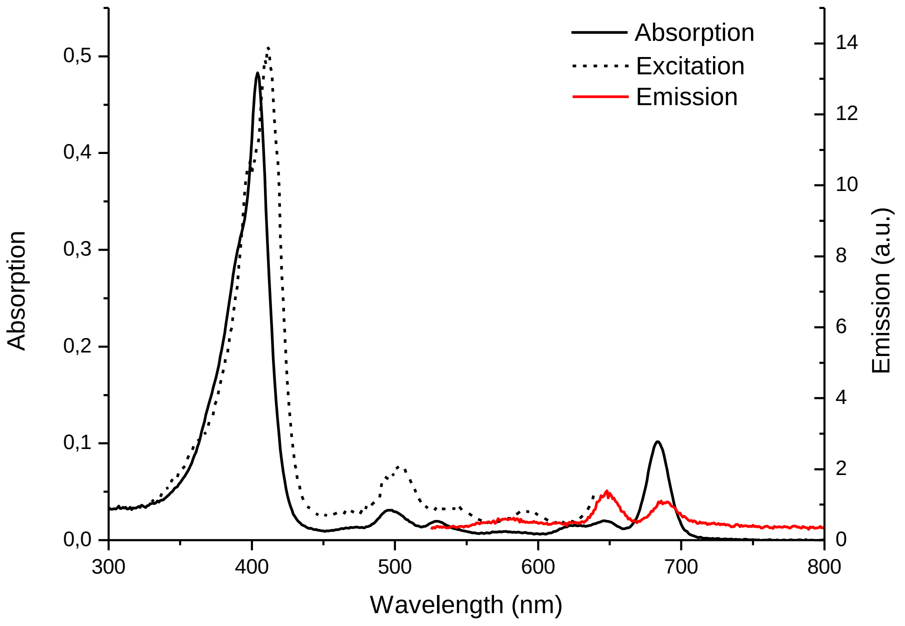

Fig. 15. Absorption, excitation (λ_{em} = 650 nm) and emission spectra (λ_{ex} = 515 nm) of compound **6a** ($2.3x10^{-6}$ mol.L^{-1} in $CHCl_3$).

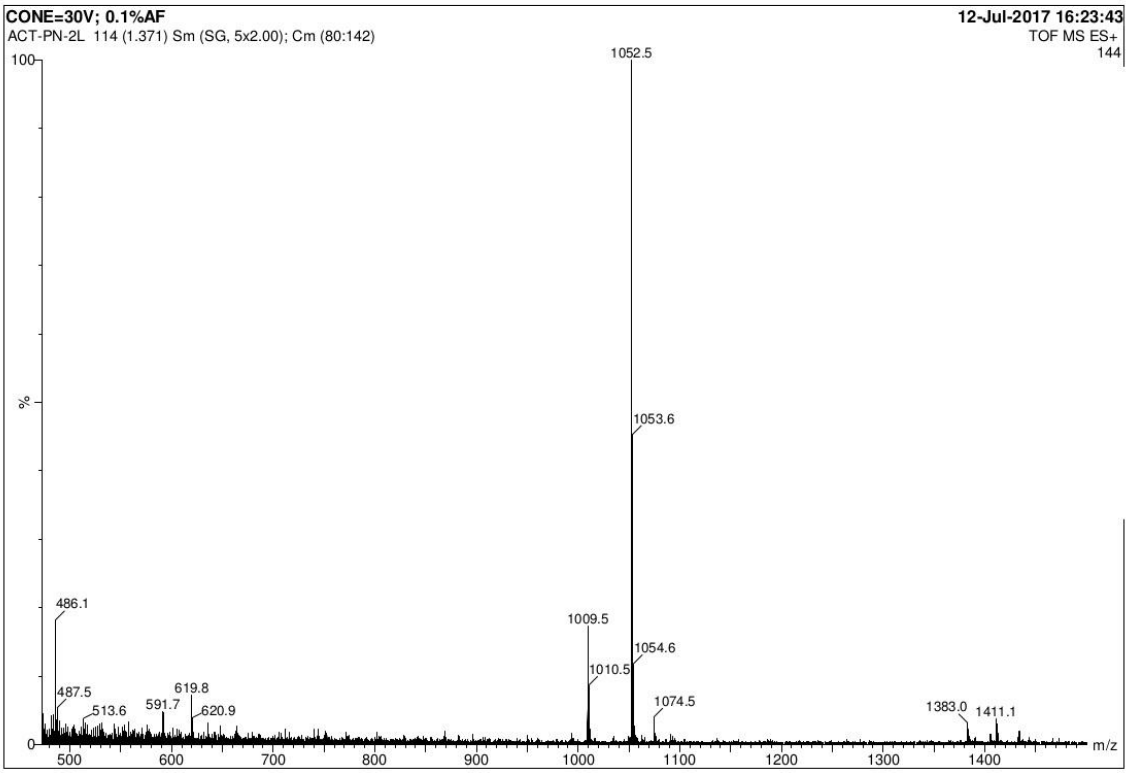

Fig. 16. Mass spectrum of the nitrone cycloadduct **6a**.

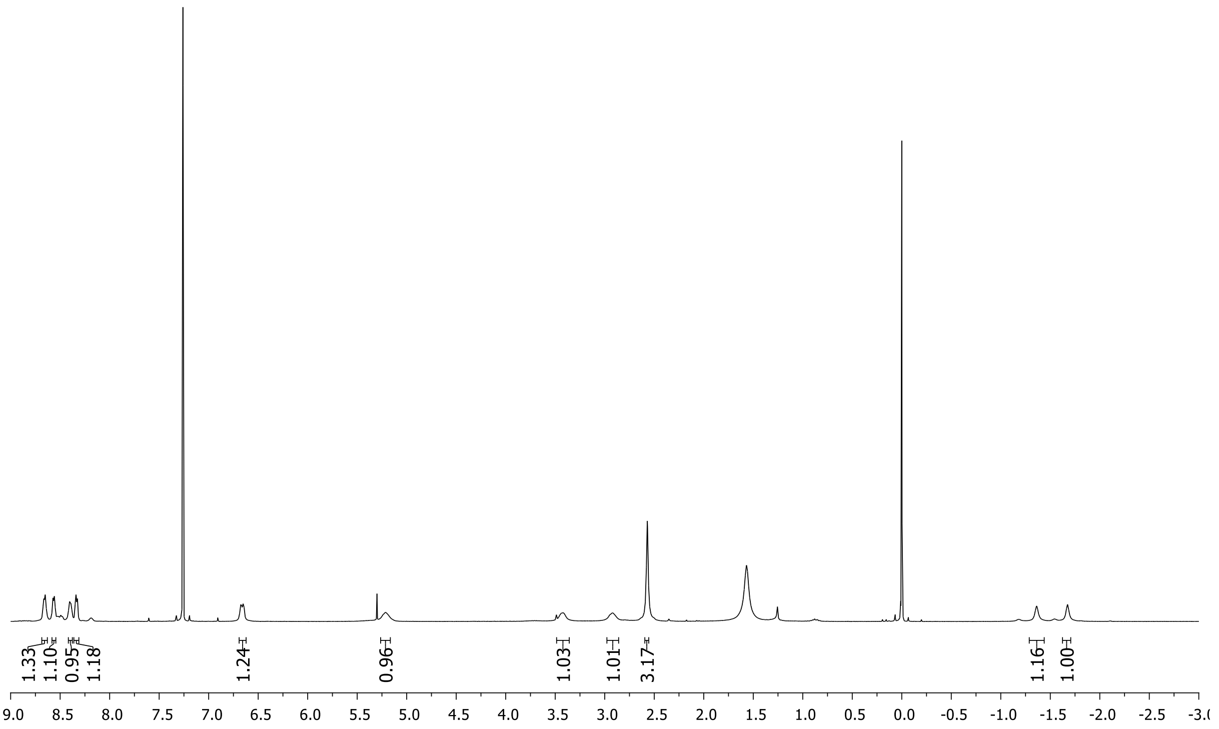

Fig. 17. ^{1}H NMR spectrum of the nitrone cycloadduct **7a** (300 MHz, $CDCl_3$).

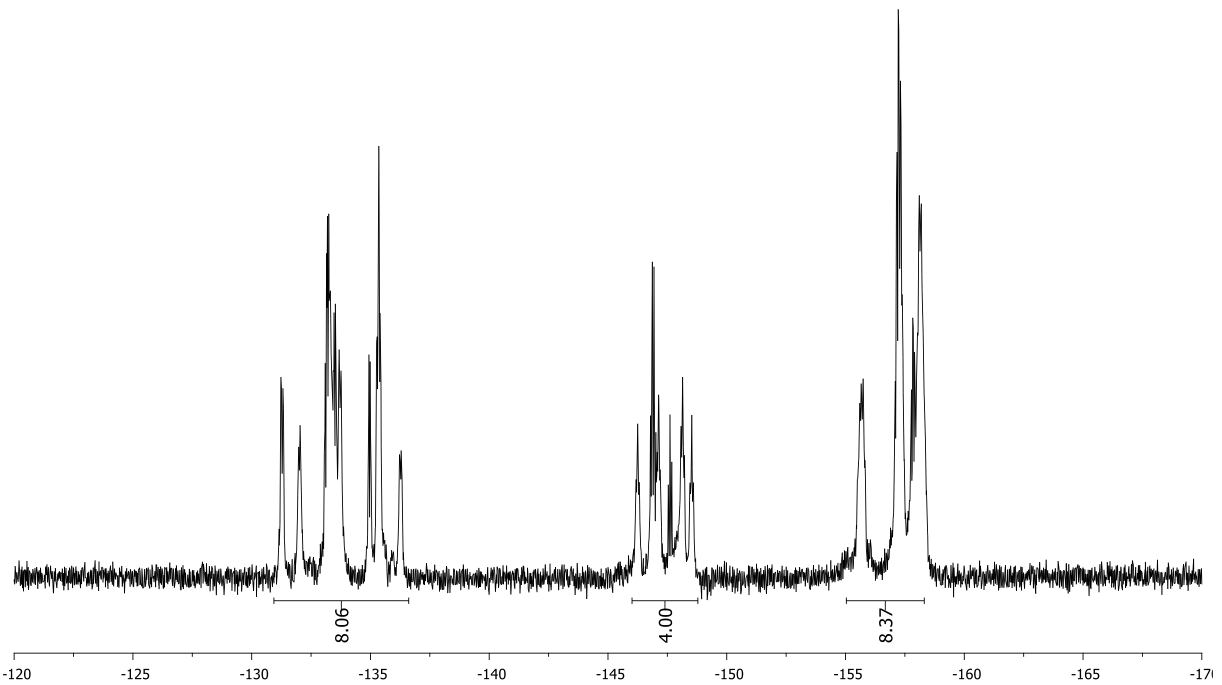

Fig. 18. ^{19}F NMR spectrum of the nitrone cycloadduct **7a** (282 MHz, $CDCl_3$).

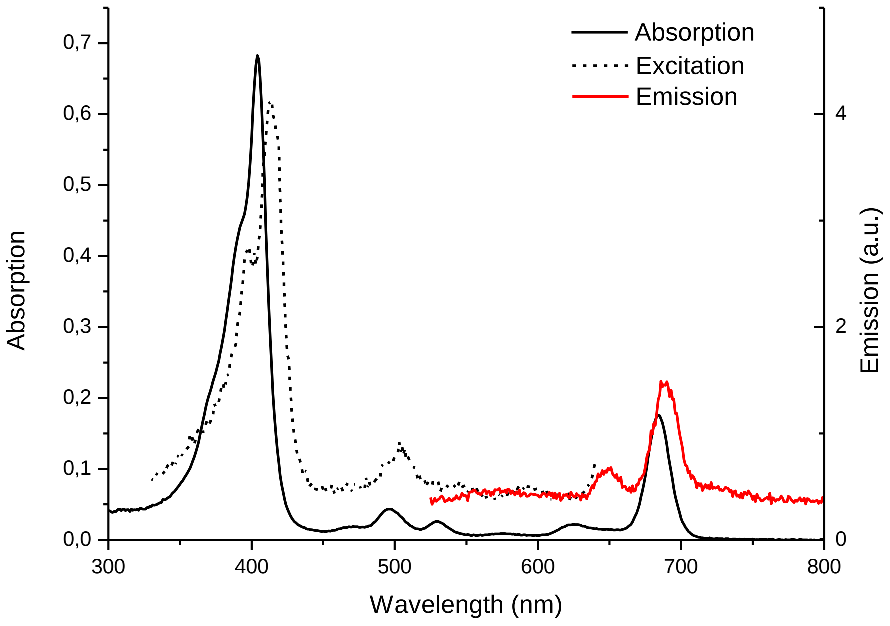

Fig. 19. Absorption, excitation (λ_{em} = 650 nm) and emission spectra (λ_{ex} = 515 nm) of compound **7a** ($2.4x10^{-6}$ mol.L^{-1} in $CHCl_3$).

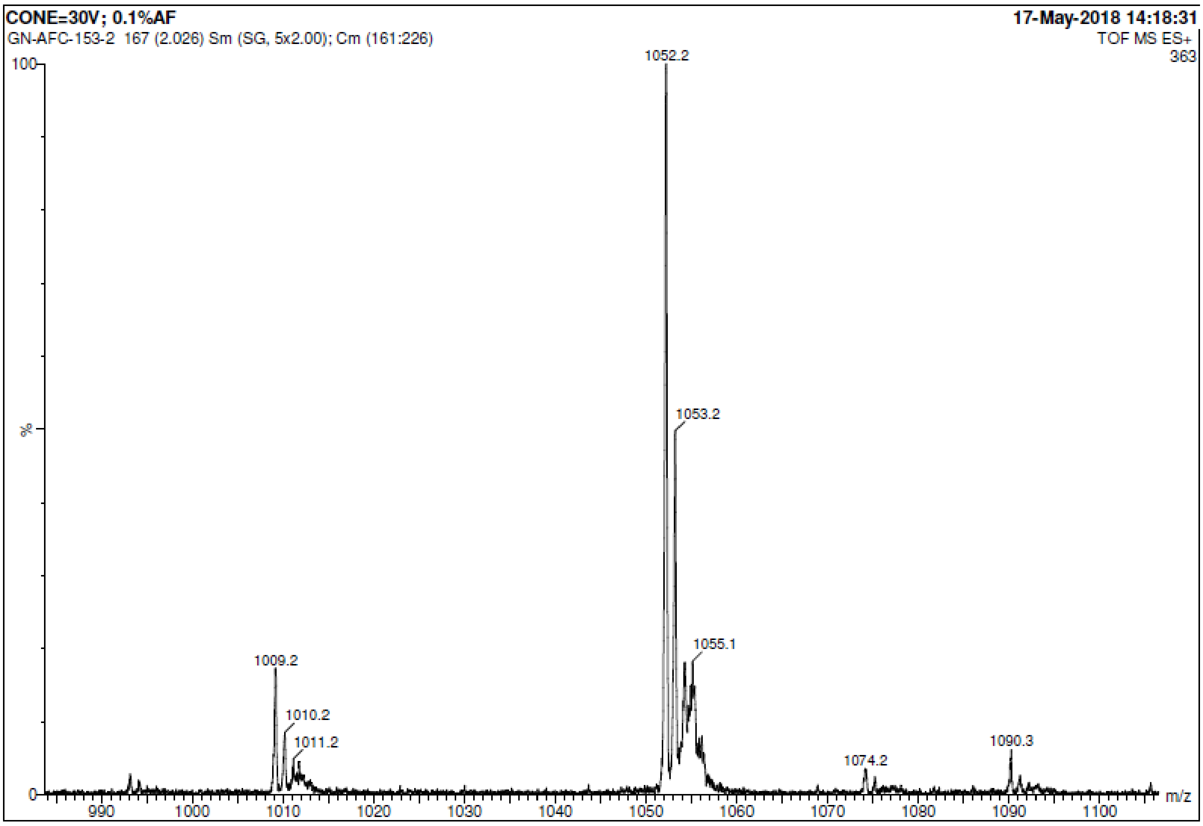

Fig. 20. Mass spectrum of the nitrone cycloadduct **7a**.

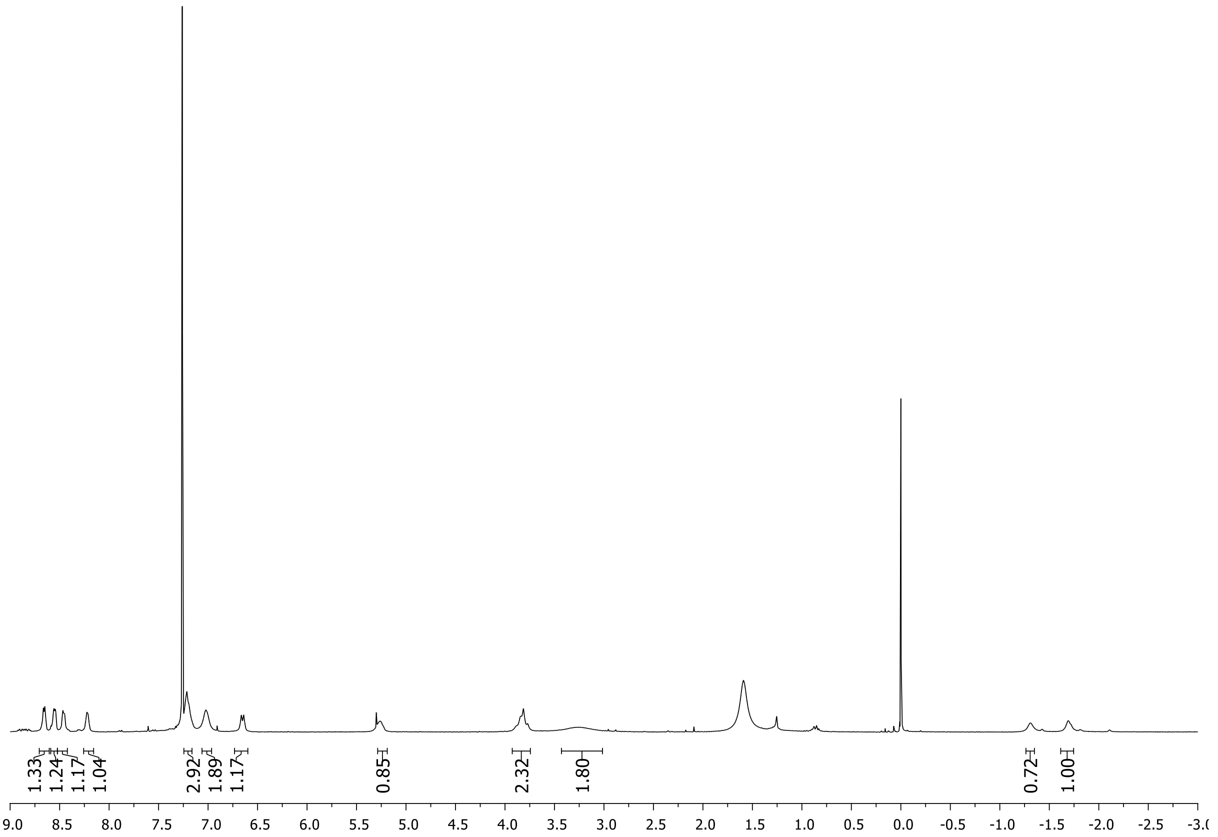

Fig. 21. ^{1}H NMR spectrum of the nitrone cycloadduct **6b** (300 MHz, $CDCl_3$).

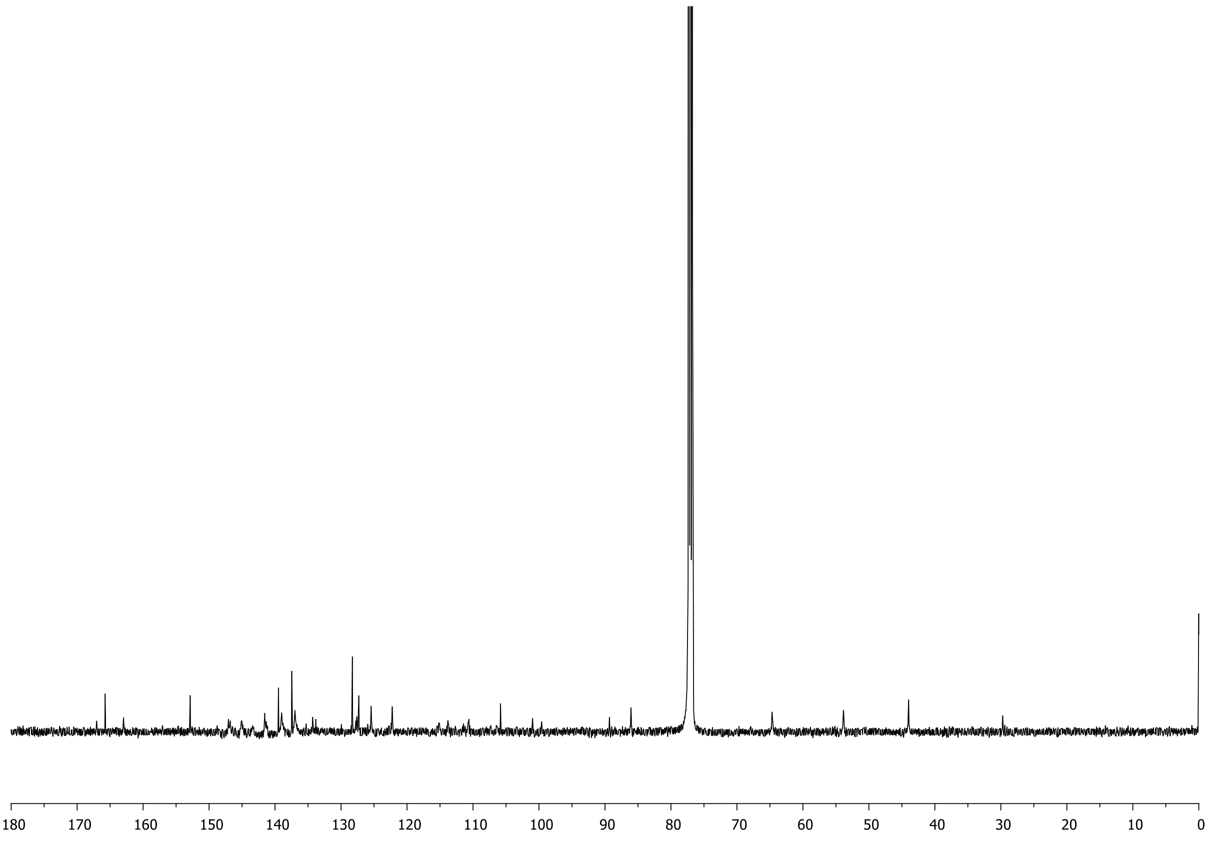

Fig. 22. ^{13}C NMR spectrum of the nitrone cycloadduct **6b** (125 MHz, $CDCl_3$).

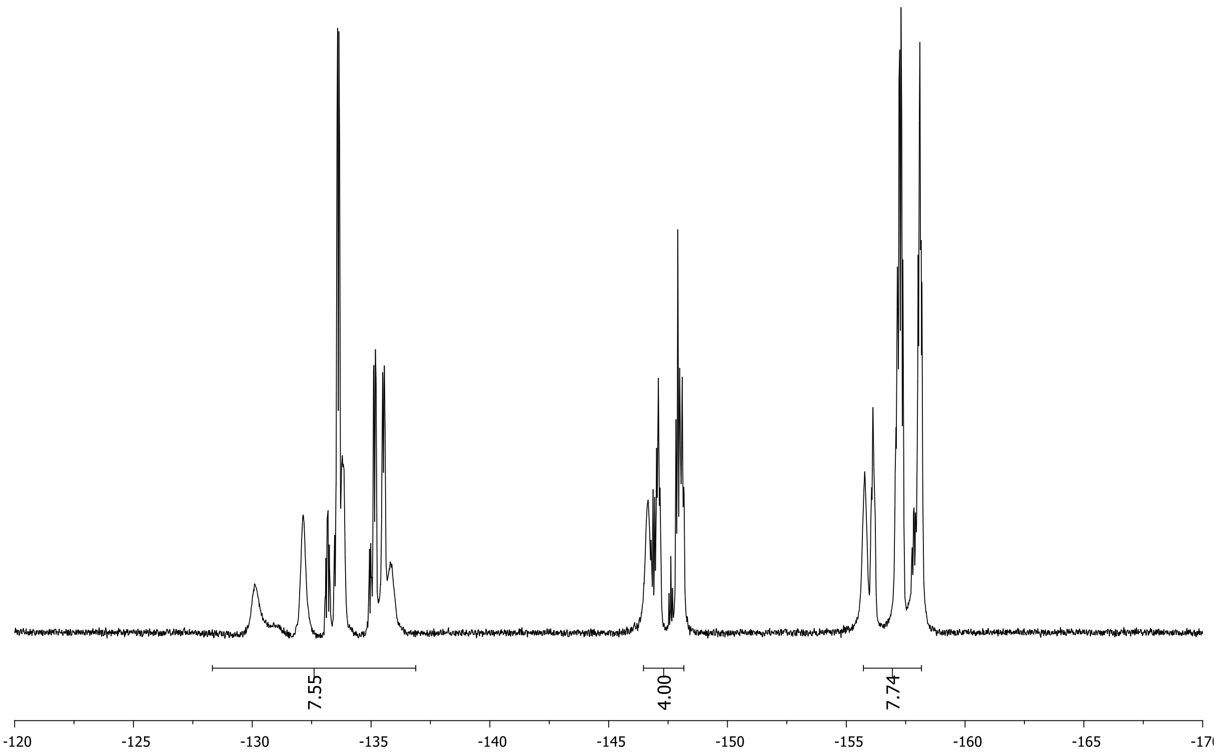

Fig. 23. ^{19}F NMR spectrum of the nitrone cycloadduct **6b** (282 MHz, $CDCl_3$).

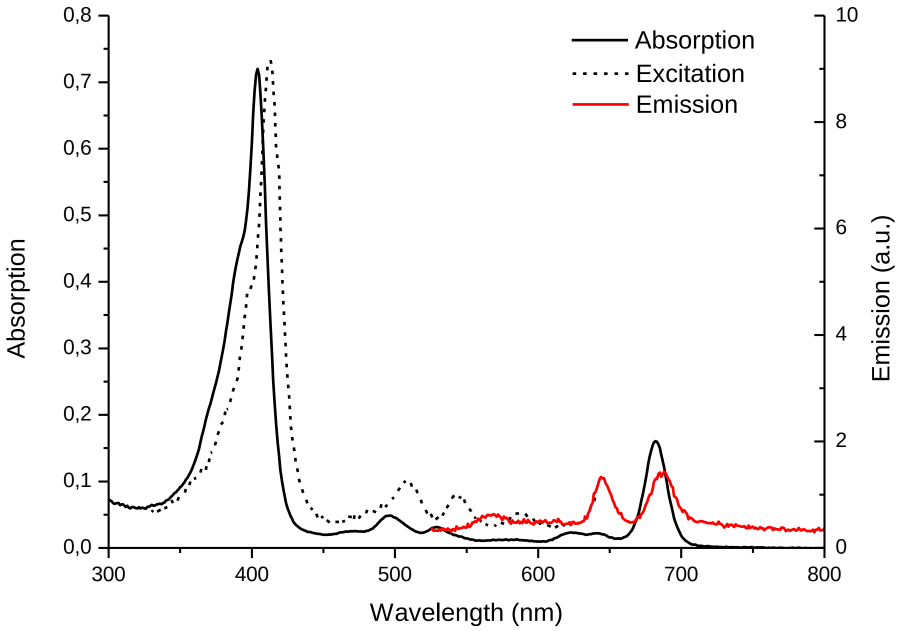

Fig. 24. Absorption, excitation (λ_{em} = 650 nm) and emission spectra (λ_{ex} = 515 nm) of compound **6b** (1.6×10^{-6} mol.L^{-1} in $CHCl_3$).

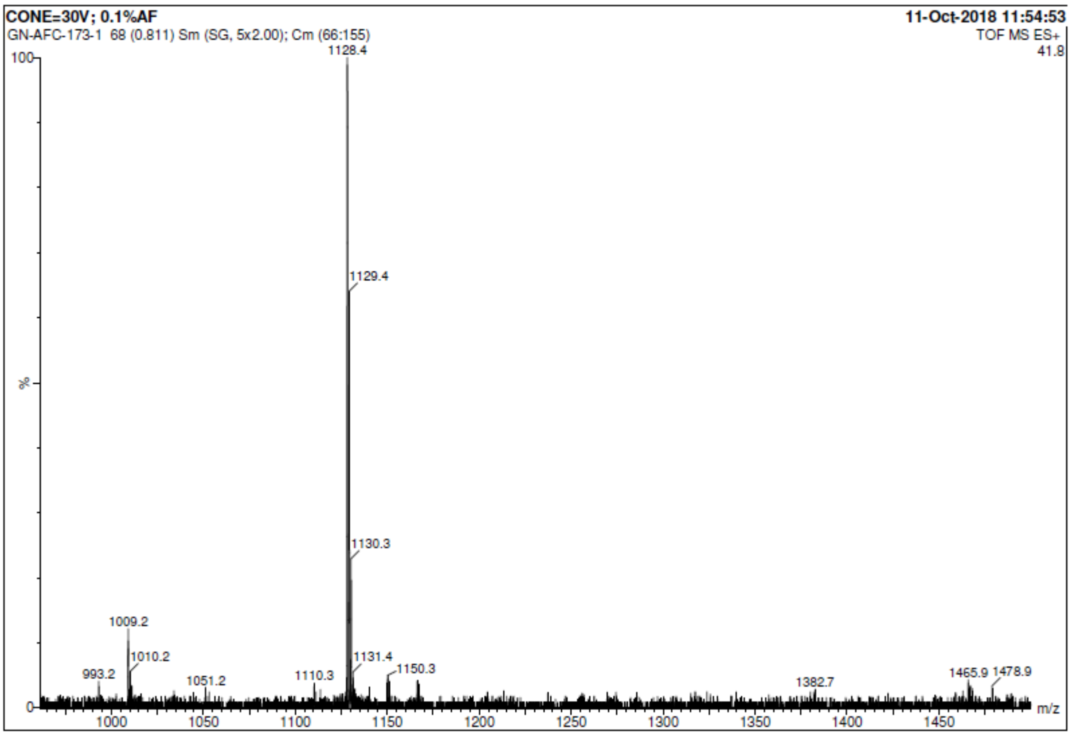

Fig. 25. Mass spectrum of the nitrone cycloadduct **6b**.

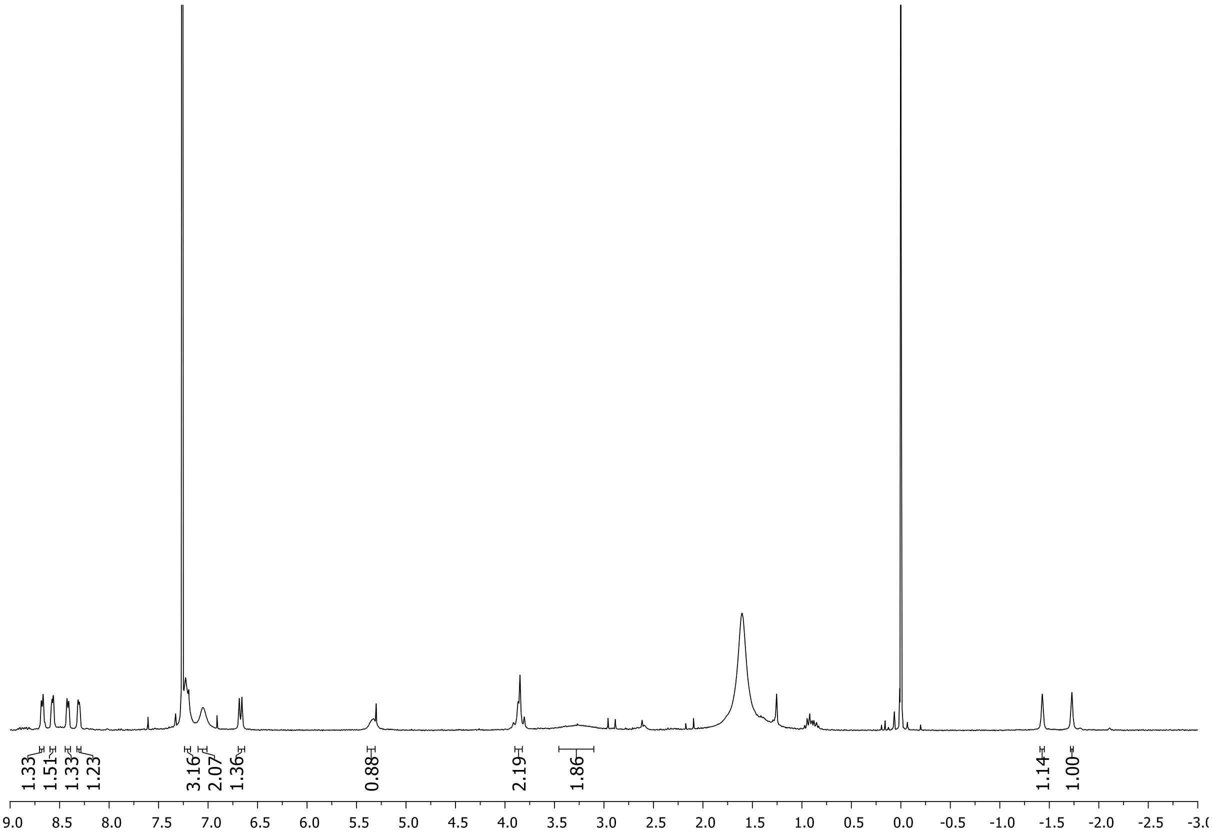

Fig. 26. ^{1}H NMR spectrum of the nitrone cycloadduct **7b** (300 MHz, $CDCl_3$).

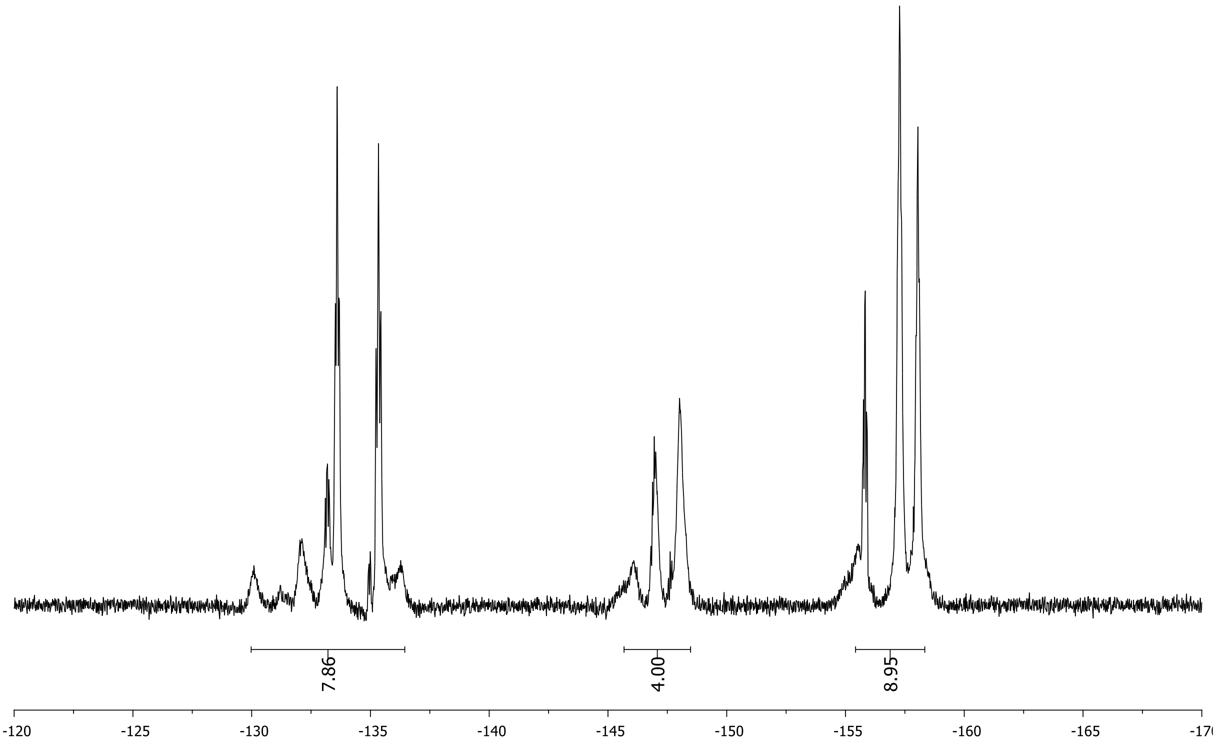

Fig. 27. ^{19}F NMR spectrum of the nitrone cycloadduct **7b** (282 MHz, $CDCl_3$).

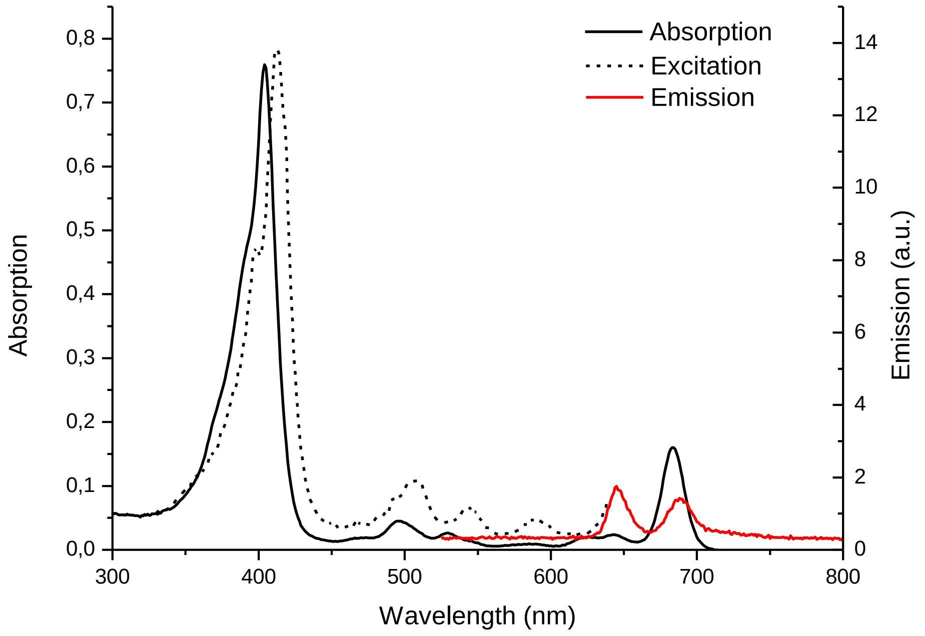

Fig. 28. Absorption, excitation (λ_{em} = 650 nm) and emission spectra (λ_{ex} = 515 nm) of compound **7b** ($1.9x10^{-6}$ $mol.L^{-1}$ in $CHCl_3$).

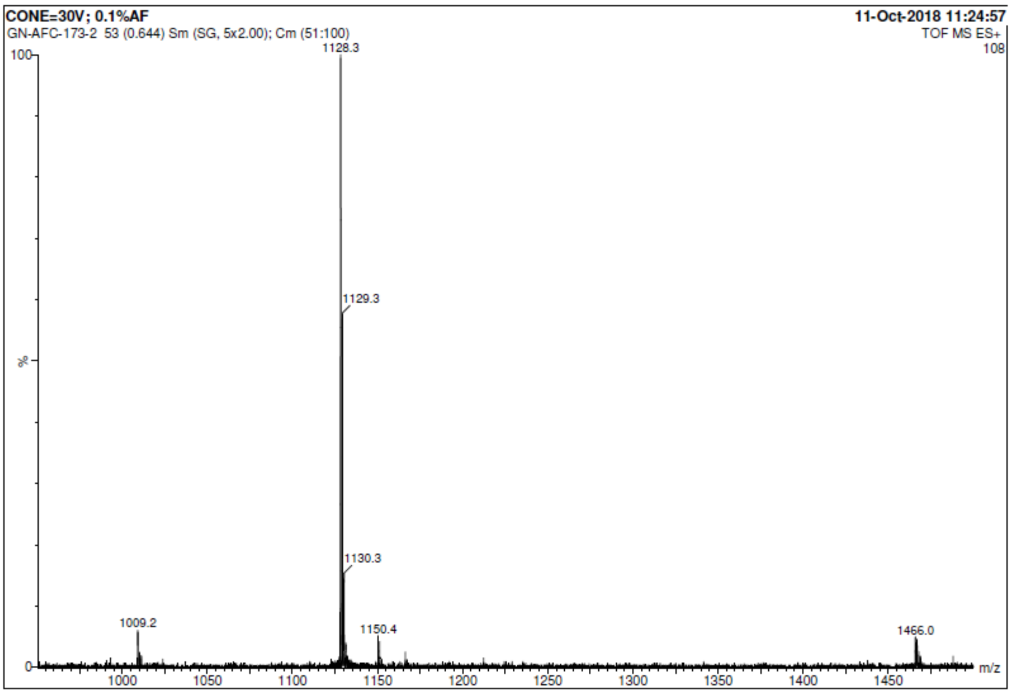

Fig. 29. Mass spectrum of the nitrone cycloadduct **7b**.

Photophysical studies

Table S1. Absorption and emission data of **3a**,**b**, **6a**,**b**, **7a**,**b** and **TPP** (standard) in chloroform.

Compounds	Absorption						Emission	
	λ_{max} (nm) (log ε, M^{-1} cm^{-1})						λ_{max}(nm)	φ_F[a]
**TPP**	418<br>(5.59)	514<br>(4.30)	547<br>(4.03)	590<br>(3.91)	647<br>(3.82)		652<br>717	0.11[b]
**3a**	382<br>(5.23)	531<br>(4.38)	572<br>(4.60)	617<br>(3.93)			582<br>629	0.59
**3b**	382<br>(5.21)	532<br>(4.34)	572<br>(4.59)	629<br>(3.56)			581<br>628	0.69
**6a**	404<br>(5.30)	497<br>(4.07)	530<br>(3.86)	625<br>(3.75)	--	684<br>(4.61)	648<br>688	0.23
**7a**	404<br>(5.44)	496<br>(4.20)	530<br>(3.97)	625<br>(3.90)	--	684<br>(4.83)	651<br>693	0.18
**6b**	404<br>(5.92)	496<br>(4.66)	529<br>(4.46)	623<br>(4.35)	646<br>(4.33)	682<br>(5.24)	644<br>688	0.15
**7b**	404<br>(5.57)	497<br>(4.30)	529<br>(4.06)	626<br>(3.97)	643<br>(4.05)	684<br>(4.87)	645<br>690	0.14

[a] Excitation at 515 nm, comparison with TPP (quantum yield 0.11 in $CHCl_3$).

[b] Boscencu, R.; Oliveira, A.S.; Ferreira, D.P.; Ferreira, L.F.V. *Int. J. Mol. Sci.* **2012**, *13*, 8112-8125.

HPLC analysis

Separation of nitrone cycloadducts **6a**/**7a** was performed using an Ultimate 3000 Thermo Scientific HPLC system. The sample was directly injected into a LiChrospher® 100 RP18 HPLC column (l = 25 cm x I.D. = 4.6 mm, 5 µm). Separation occurred using isocratic elution with a mixture of 30% buffer A (1% formic acid in water) and 70% buffer B (1% formic acid in acetonitrile) over 25 minutes at a flow rate of 1.0 $mL.min^{-1}$. Buffer B was increased to 100% in 5 min and maintained until 44 min. Column temperature was kept at 40 °C.

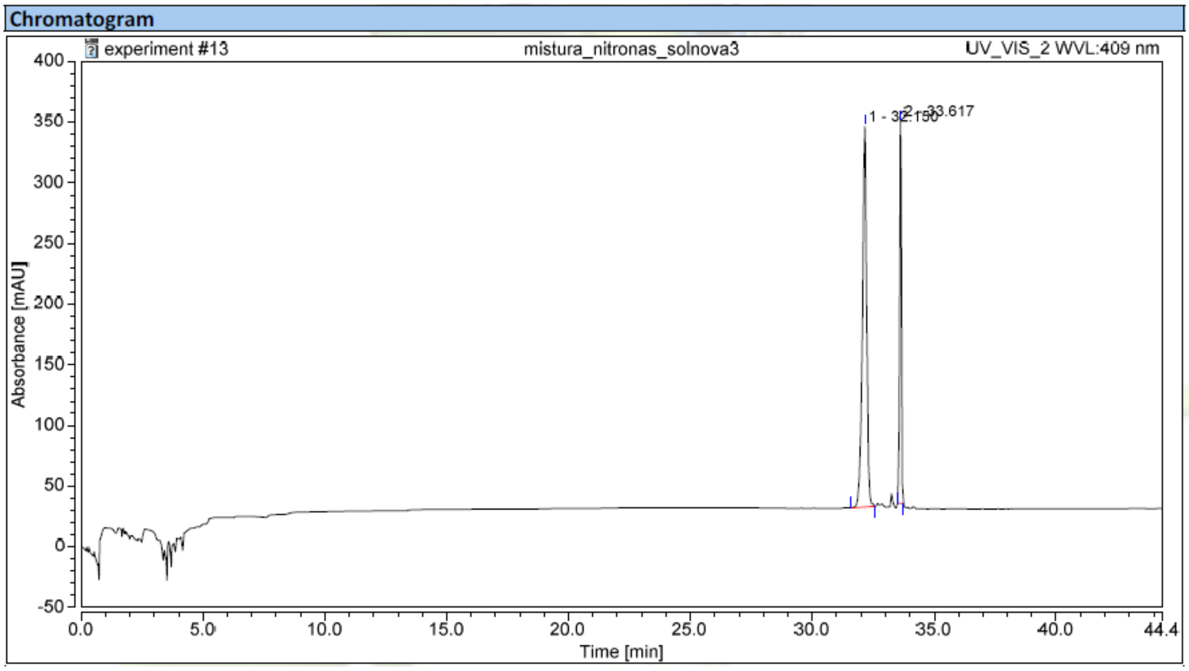

Fig. 30. HPLC analysis (at λ = 409 nm) of a ~1:1 mixture of cycloadducts **6a** and **7a**: peak 1 (retention time (RT) = 32.15 min) corresponds to the minor isomer (**7a**) and peak 2 (RT = 33.62 min) corresponds to the major isomer (**6a**).

Single crystal X-ray diffraction studies

Single crystals of compounds **3b** and **6a** were manually harvested from the crystallization vials and immersed in highly viscous FOMBLIN Y perfluoropolyether vacuum oil (LVAC 140/13, Sigma-Aldrich) to avoid degradation caused by the evaporation of the solvent.[1] Crystals were mounted on either Hampton Research CryoLoops or MiTeGen MicroLoops, typically with the help of a Stemi 2000 stereomicroscope equipped with Carl Zeiss lenses.

X-ray diffraction data for compound **6a** were collected at 150(2)K on a Bruker D8 QUEST equipped with Mo Kα sealed tube (λ = 0.71073 Å), a multilayer TRIUMPH X-ray mirror, a PHOTON 100 CMOS detector, and an Oxford Instruments Cryostrem 700+ Series low temperature device.

Crystal data for **3b** were instead collected at 150(2)K on a Bruker X8 Kappa APEX II CCD area-detector diffractometer (Mo Kα graphite-monochromated radiation, λ = 0.71073 Å) controlled by the APEX3 software package[2] and equipped with an Oxford Cryosystems Series 700 cryostream monitored remotely using the software interface Cryopad.[3] In both cases, diffraction images were processed using the software package SAINT+,[4] and data were corrected for absorption by the multiscan semi-empirical method implemented in SADABS 2016/2.[5]

Structures were solved using the algorithm implemented in SHELXT-2014/5,[6] which allowed the immediate location of almost all of the heaviest atoms composing the molecular unit of the compounds. The remaining missing and misplaced non-hydrogen atoms were located from difference Fourier maps calculated from successive full-matrix least-squares refinement cycles on F^2 using the latest SHELXL from the 2018/3 release.[7] All structural refinements were performed using the graphical interface ShelXle.[8]

Hydrogen atoms bound to carbon and nitrogen were placed at their idealized positions using appropriate *HFIX* instructions in SHELXL: *43* (aromatic and vinylic carbon atoms), *13* (tertiary carbon atoms), *23* (–CH_2– carbon atoms), and *137* (for terminal methyl groups). These hydrogen atoms were included in subsequent refinement cycles with isotropic thermal displacements parameters (U_{iso}) fixed at 1.2 or 1.5×U_{eq} (for the former family of hydrogen atoms) of the parent non-hydrogen atoms.

Structural drawings have been created using the software package Crystal Impact Diamond.[9]

The last difference Fourier map synthesis showed for **6a** the highest peak (1.254 eÅ^{-3}) and the deepest hole (-0.529 eÅ^{-3}) located at 1.17 and 0.58 Å from N5 and C301, respectively. This may suggest some level of structural disorder of the moieties associated with the cycloaddition but after structural refinement as for **3b** the partial occupancies were too low to be considered as significant.

Crystallographic data for the structures reported in this paper have been deposited with the Cambridge Crystallographic Data Centre as supplementary publication Noa. CCDC-1832879, 1832880 and 1985131. Copies of the data can be obtained free of charge on application to CCDC, 12 Union Road, Cambridge CB2 2EZ, U.K. FAX: (+44) 1223 336033. E-mail: deposit@ccdc.cam.ac.uk.

---

1. Kottke, T.; Stalke, D., *J. Appl. Crystallogr.* **1993,** *26*, 615-619.

2. APEX3, *Data Collection Software Version 2016.9-0, Bruker AXS, Delft, The Netherlands* **2005-2016**.

3. Cryopad, *Remote monitoring and control, Version 1.451, Oxford Cryosystems, Oxford, United Kingdom* **2006**.

4. SAINT+, *Data Integration Engine v. 8.37a*© **1997-2015**, Bruker AXS, Madison, Wisconsin, USA.

5. Krause, L.; Herbst-Irmer, R.; Sheldrick, G. M.; Stalke, D., Comparison of silver and molybdenum microfocus X-ray sources for single-crystal structure determination. *J. Appl. Crystallogr.* **2015,** *48*, 3-10.

6. Sheldrick, G. M., SHELXT - Integrated space-group and crystal-structure determination. *Acta Cryst. A* **2015,** *71*, 3-8.

7. Sheldrick, G. M., Crystal structure refinement with SHELXL. *Acta Cryst. C* **2015,** *71*, 3-8.

8. Hübschle, C. B.; Sheldrick, G. M.; Dittric, B., *J. Appl. Crystallogr.* **2011,** *44*, 1281-1284.

9. Brandenburg, K., *DIAMOND, Version 3.2f. Crystal Impact GbR, Bonn, Germany* **1997-2010**.
